# Specificity and Versatility of Substrate Binding Sites in Four Catalytic Domains of Human N-Terminal Acetyltransferases

**DOI:** 10.1371/journal.pone.0052642

**Published:** 2012-12-28

**Authors:** Cédric Grauffel, Angèle Abboud, Glen Liszczak, Ronen Marmorstein, Thomas Arnesen, Nathalie Reuter

**Affiliations:** 1 Department of Molecular Biology, University of Bergen, Bergen, Norway; 2 Computational Biology Unit, Uni Computing, Uni Research AS, Bergen, Norway; 3 The Wistar Institute, University of Pennsylvania, Philadelphia, Pennsylvania, United States of America; 4 Department of Chemistry, University of Pennsylvania, Philadelphia, Pennsylvania, United States of America; 5 Department of Surgical Sciences, University of Bergen, Bergen, Norway; King’s College, London, United Kingdom

## Abstract

Nt-acetylation is among the most common protein modifications in eukaryotes. Although thought for a long time to protect proteins from degradation, the role of Nt-acetylation is still debated. It is catalyzed by enzymes called N-terminal acetyltransferases (NATs). In eukaryotes, several NATs, composed of at least one catalytic domain, target different substrates based on their N-terminal sequences. In order to better understand the substrate specificity of human NATs, we investigated *in silico* the enzyme-substrate interactions in four catalytic subunits of human NATs (Naa10p, Naa20p, Naa30p and Naa50p). To date hNaa50p is the only human subunit for which X-ray structures are available. We used the structure of the ternary hNaa50p/AcCoA/MLG complex and a structural model of hNaa10p as a starting point for multiple molecular dynamics simulations of hNaa50p/AcCoA/substrate (substrate = MLG, EEE, MKG), hNaa10p/AcCoA/substrate (substrate = MLG, EEE). Nine alanine point-mutants of the hNaa50p/AcCoA/MLG complex were also simulated. Homology models of hNaa20p and hNaa30p were built and compared to hNaa50p and hNaa10p. The simulations of hNaa50p/AcCoA/MLG reproduce the interactions revealed by the X-ray data. We observed strong hydrogen bonds between MLG and tyrosines 31, 138 and 139. Yet the tyrosines interacting with the substrate’s backbone suggest that their role in specificity is limited. This is confirmed by the simulations of hNaa50p/AcCoA/EEE and hNaa10p/AcCoA/MLG, where these hydrogen bonds are still observed. Moreover these tyrosines are all conserved in hNaa20p and hNaa30p. Other amino acids tune the specificity of the S1’ sites that is different for hNaa10p (acidic), hNaa20p (hydrophobic/basic), hNaa30p (basic) and hNaa50p (hydrophobic). We also observe dynamic correlation between the ligand binding site and helix 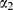 that tightens under substrate binding. Finally, by comparing the four structures we propose maps of the peptide-enzyme interactions that should help rationalizing substrate-specificity and lay the ground for inhibitor design.

## Introduction

Acetylation is one of the most common co- or post-translational protein modifications. There are two major types of acetylations: on lysine side chains (K-acetylation), and on N-terminal residues (Nt-acetylation). To date, there is far more information on K-acetylation, and especially on the responsible enzymes, the lysine acetyl-transferases (KATs), as compared to Nt-acetylation [1].

Nt-acetylation is the transfer of an acetyl group from acetyl coenzyme A (AcCoA) to the 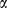-amino group of the first amino acid residue of a protein. It is among the most common protein modifications in eukaryotes, as it occurs on approximately 50–70% of yeast proteins and 80–90% of human proteins [2], [3]. Unlike lysine acetylation, N-terminal acetylation is irreversible and happens mainly co-translationally. It is catalyzed by enzymes denoted N-terminal acetyltransferases (NATs), which are associated with ribosomes [4]. The role of Nt-acetylation is currently debated. It was long thought to be a way of protecting proteins from ubiquitin-dependent degradation [5], [6]. According to recent data, the opposite may also be true since acetylated N-termini are recognized by the Doa10 ubiquitin ligase for ubiquitin-mediated degradation [7]. Furthermore, roles of Nt-acetylation in protein complex formation, membrane targeting and preventing endoplasmic reticulum translocation were recently presented [8], [9].

In eukaryotes, several NATs (from NatA to NatF) composed of at least one catalytic subunit Naa10p-Naa60p, have been identified [3]. Each type targets different substrates based on their N-terminal sequence, where the first two amino acids seem to be most decisive [10]. NatA, NatB, and NatC complexes are together responsible for most of the Nt-acetylations [11], and are conserved between yeasts and human in terms of both subunits composition and substrate specificity [4]. The NatA complex is composed of two catalytic subunits, Naa10p and Naa50p (former names ARD1 and NAT5/SAN, respectively), plus two other subunits, Naa15p (NAT1/NATH) and HYPK (for Huntingtin Yeast two-hybrid Protein K), that may be involved in ribosome and/or peptide association. Naa50p and Naa10p exhibit different substrate specificities, as the first one targets sequences containing an N-terminal Met, while the latter acetylates chains starting with Ser, Ala, Gly, Val, Cys, Thr, Asp or Glu [2], [12], [13].

Structural data on human NATs and their catalytic subunits is still scarce. A few structures of GNAT acetyltransferases have been solved by X-ray diffraction (2JDC from *Bacillus Licheniformis* [14], 3GUW from *Bordetella Pertussis*, unpublished). The first structure of a catalytic domain of a human NAT (hNaa50p) was recently resolved in the presence of a 10-mer peptide and CoA (PDBID : 3TFY). The first four amino-acids of the peptide substrate (Met-Leu-Gly-Pro) are resolved and provide the molecular basis for the substrate specificity of hNaa50p. The side-chains of Met1 and Leu2 form van der Waals interactions with the hNaa50p residues in the hydrophobic pocket, and further, a number of hydrogen bonds anchors the N-terminal peptide to the enzyme. The substrate pocket is more constricted than similar binding sites found in KATs, explaining why hNaa50p strongly prefers N-terminal substrates to lysine side chains. Several of the hNaa50p residues responsible for the peptide backbone interactions are also conserved in hNaa10p, suggesting a conserved NAT-mode of binding to the N-terminal peptides [15]. In order to further shed light on the structures of the NAT-enzymes, and in particular the ability of different NATs to N-terminally acetylate distinct substrate-classes, we applied a computational approach to investigate the substrate specificity of hNaa50p and three other catalytic subunits of human NATs (10p, 20p and 30p).

Taking advantage of the X-ray structure of the hNaa50p/CoA/MLGP complex, we ran multiple 10 nanoseconds long molecular dynamics (MD) simulations of hNaa50p/AcCoA/MLG, hNaa50p/AcCoA/MKG and hNaa50p/AcCoA/EEE. The first two peptide sequences (MLG and MKG) are known to be efficiently acetylated by hNaa50p while the third one (EEE) is rather a hNaa10p substrate [12]. Simulations of the three complexes provide a detailed picture of the enzyme-peptide interactions and form the basis to further explain the substrate peptide sequence selectivity of hNaa50p. We also investigated the influence of the ligands on the enzyme flexibility and the dynamical cross-correlation. Simulations of the hNaa50p complexes are complemented by simulations of nine single point alanine mutants of hNaa50p/AcCoA/MLG. Further, we built homology models for hNaa10p and performed simulations of hNaa10p/AcCoA/MLG and hNaa10p/AcCoA/EEE. Finally, we built similar homology models using the catalytic subunits of NatB and NatC, hNaa20p and hNaa30p, respectively. Taken together and discussed in the context of available experimental data, our results allow us to propose a model of the structural basis for the specificity of the catalytic hNATs subunits.

## Results and Discussion

**Figure 1:**
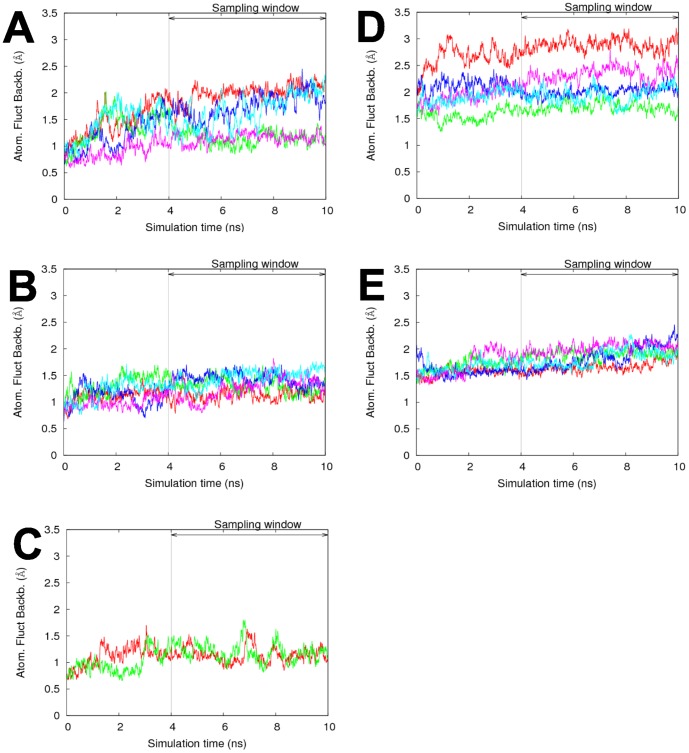
Backbone RMSD (in Å) along MD simulations. A, B, C: RMSD of hNaa50p backbone with MLG, EEE and in the *apo* form, respectively. D, E: RMSD of hNaa10p backbone with MLG and EEE substrates, respectively. Each line corresponds to a replicate (same starting structure but different velocities distributions).

In order to better understand the interactions governing the specificity of the catalytic domains of human NatA and NatF, we performed 10 ns-long molecular dynamics simulations of hNaa50p, both of the free enzyme with AcCoA, and with three different peptides docked into the active site, and of both the wild type form and nine different mutants. We built a structural model of hNaa10p which we simulated with two different substrates. To ensure sufficient sampling, all simulations were run at least twice (Cf. Table 1, Methods section). RMSd variation along simulation time shows that there is no large change of backbone conformation in any of the systems simulated and that the simulations are stable over the last 6 ns (see Figure 1 and Figure S1 of Supp. Mat.). We analyzed the 44 simulation trajectories with a particular focus on the distance between N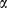 and AcCoA, hydrogen bonding patterns, free energy decompositions and quasi-harmonic modes. The results pertinent to the understanding of the ligand-enzyme interactions are presented below.

**Table 1:** Number of simulations performed for each system.

Domain	Mutation	Peptide	Number of Simulations
hNaa50p	no	MLG	5
	F27A	MLG	2
	P28A	MLG	2
	V29A	MLG	2
	Y31A	MLG	2
	F35A	MLG	2
	Y73A	MLG	2
	H112A	MLG	2
	Y139A	MLG	2
	I142A	MLG	2
	no	EEE	5
	no	MKG	2
	no	no	2
	F27A	no	2
hNaa10p	no	EEE	5
	no	MLG	5

### Effect of Ligands on the Structure and Dynamics of hNaa50p

#### Ligand-enzyme interactions in hNaa50p/AcCoA/MLG and hNaa50p/AcCoA/MKG

**Figure 2:**
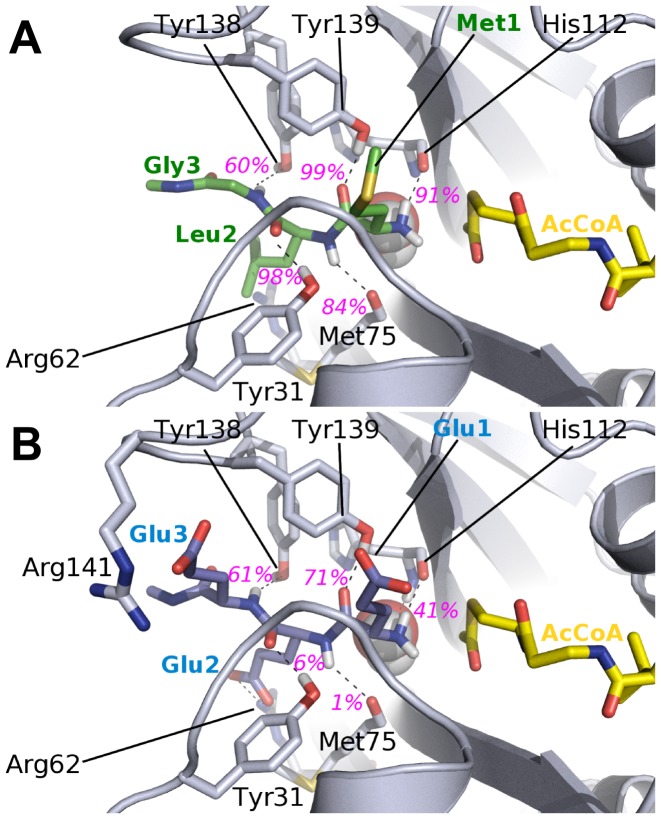
Substrate binding site of MLG (A) and EEE (B) peptides in hNaa50p. The peptides (MLG in green, EEE in blue), the side chains of amino acids (in grey) interacting with them, as well as the co-factor AcCoA (in yellow) are represented with sticks. The crystallographic water W is represented with spheres. The average lifetimes of the hydrogen bonds (dashed lines) involving peptide’s backbone during the last 6 ns of simulation are labelled in magenta.

**Figure 3:**
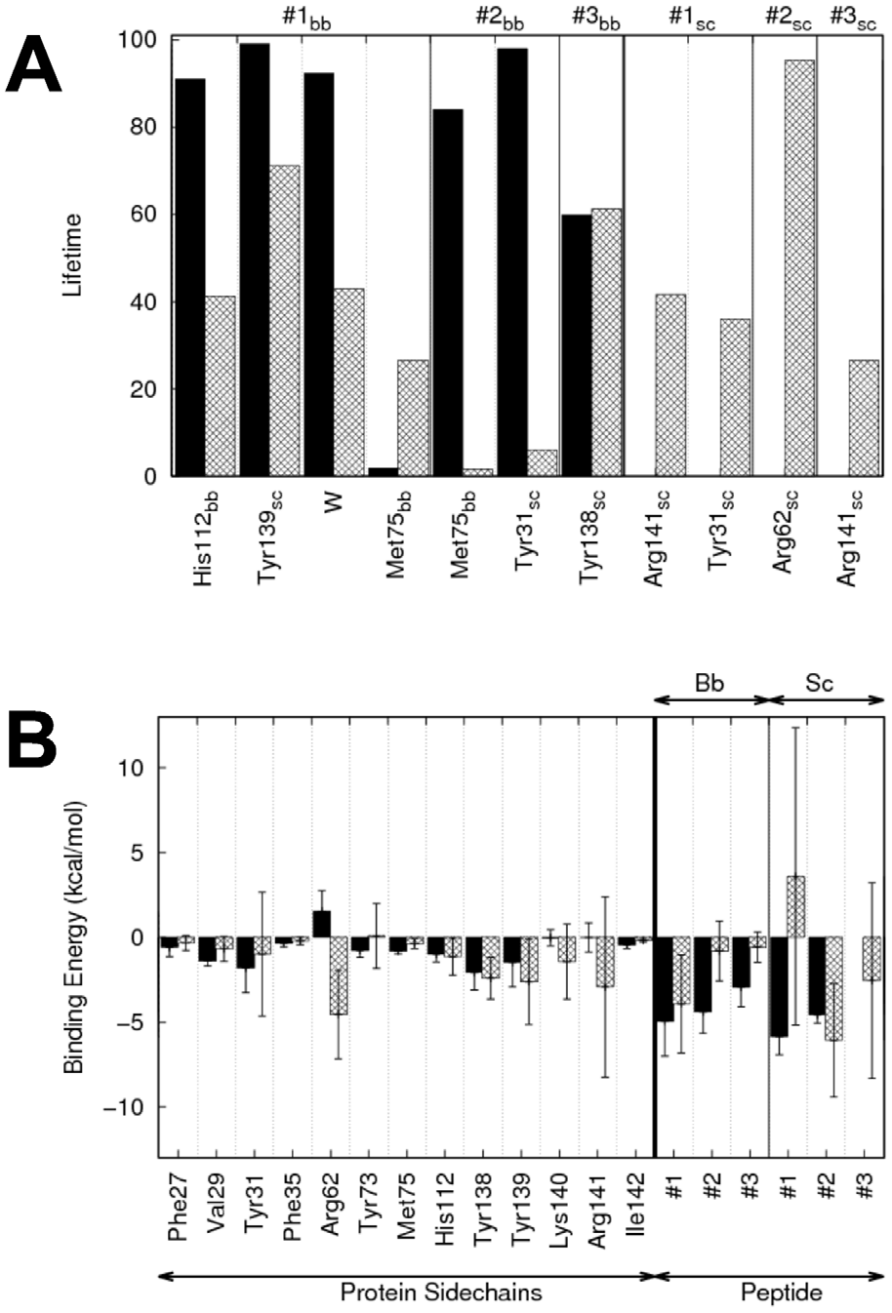
Hydrogen bonds lifetimes and contributions to the free energy of binding in hNaa50p. A: Average hydrogen bonds lifetimes during the last 6 ns of the simulations of hNaa50p with MLG (black bars) and EEE (grey) peptides. “bb” and “sc” correspond to backbone or side chain of the corresponding residue, respectively. #1, #2 and #3 are the peptide residue numbers. B : Amino acid residues of hNaa50p/MLG (black) and hNaa50p/EEE (grey) with the highest contribution to the free energy of binding (MM/PBSA). The contributions are divided between backbone (bb) and side chains (sc) contributions.

We manually built the acetyl group on CoA since the X-ray structure was determined with CoA (and not AcCoA) [15] (Cf. Methods section). We also had to define a number of new force field parameters for the cofactor (described in Methods section). Hence it is important to compare the results of the simulations of hNaa50p/AcCoA/MLG against the X-ray structure of hNaa50p/CoA/MLGP to verify the validity of the starting structure we used and of the force field parameters. The average distance between the N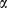 of Met1 and the acetyl group of AcCoA measured over five independent 10 ns-simulations is 3.3 Å (Cf. Table 2) and is very stable in all simulations indicating a good positioning of the substrate and AcCoA in the starting structure. The X-ray structure of hNaa50p (PDBID : *3TFY*) shows a MLGP peptide stabilized at the surface of the enzyme by several hydrogen bonds to its backbone. Three of these are mediated by the hydroxyl groups of tyrosines 139, 31 and 138. In agreement with the X-ray data, we observe in our MD simulations of hNaa50p with MLG strong hydrogen bonds between Tyr139 and the carbonyl group of Met1, and between Tyr31 and the carbonyl groups of Leu2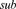 (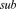 is used to differentiate amino acids of the substrates from enzyme residues). Tyr138 interacts less strongly with the NH group of Gly3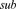 (Cf. Figures 2A and 3A). The first two hydrogen bonds are present most of the simulation time (hydrogen bond life-time of 99% and 97%, respectively) and indicate very strong interactions while the third one is present in only about 65% of the conformations collected during the simulation. Backbone-backbone hydrogen bonds between the peptide and amino acids His112 and Met75 have also been described [15]. Both of these are observed in the simulations and with long life-times. The hydrogen bond between the carbonyl group of His112 and the N-terminus of the peptide is somewhat stronger than Met75-Leu2. An additional stabilization is made by a water molecule, which is bridging the N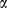 of Met1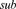, Ile74 and His112. Consistently, free energy decomposition using the MM/PBSA method (Figure 3B) yield very favorable binding energies for the backbone of the peptide, especially for residues Met1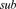 and Leu2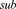. The side chains of these two hydrophobic residues are also involved in extensive van der Waals interactions with the protein that yield high contributions to the binding free energy. Among protein residues, we observe smaller binding energies; Tyr138 and Tyr139 contribute the most via van der Waals interactions. The side chains of Val29, Tyr73, Met75 and His112 exhibit a smaller contribution. Arg62, which is facing Leu2, is the only residue with an unfavorable binding energy, but this small electrostatic repulsion could play a role in pushing the substrate toward the AcCoA.

**Table 2:** Distances between the N-terminal nitrogen atom of the peptides and the carbonyl carbon of Ac-CoA.

Domain	Mutation	Peptide	Dist. (Å)
7*hNaa50p	no	MLG	3.3 ± 0.2
	F27A	MLG	4.0 ± 1.0
	P28A	MLG	3.8 ± 0.3
	V29A	MLG	3.5 ± 0.4
	Y31A	MLG	3.4 ± 0.3
	F35A	MLG	3.4 ± 0.2
	Y73A	MLG	3.3 ± 0.2
	H112A	MLG	3.6 ± 0.4
	Y139A	MLG	3.5 ± 0.4
	I142A	MLG	3.7 ± 0.5
	no	M**K**G	3.3 ± 0.2
	no	EEE	5.2 ± 2.2
2*hNaa10p	no	EEE	4.2 ± 0.6
	no	MLG	5.9 ± 1.9

The values are averaged over each simulation.

Our simulations depict a complex stabilized through strong hydrogen bonding of the enzyme to the backbone of the peptide, and van der Waals interactions via side chains of both the peptide and the enzyme. These interactions are significantly stronger with the first two amino acids of the substrate than with the third (Gly3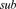). This is in agreement with X-ray diffraction data of the complex between hNaa50p and MLGP showing that B factors increase from Met1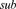 to Pro4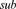 [15]). The three tyrosine amino acids provide robust anchoring points to the peptide backbone and we do not expect them to be responsible for the specificity of hNaa50p. Because of their hydrophobic and polar character, they can actually accommodate a rather wide range of amino acid types. We believe that the tyrosines need to be supplemented by additional interactions between the enzyme and the peptide side chains to achieve specificity.

To investigate this hypothesis, we ran simulations of hNaa50p with MKG, a sequence experimentally shown to be efficiently acetylated by the enzyme [12]. Indeed according to Van Damme & al, hNaa50p could also specifically acetylate peptides having Met-Lys, Met-Ala and Met-Met N-termini. Interestingly the MKG peptide possesses a positively charged residue in P2’ which is very different from the hydrophobic leucine of MLG. The simulations showed limited differences between the complexes with MLG and MKG, both in terms of dynamics and ligand-enzyme interactions. The stability of the hydrogen bonds mediated by the backbone is similar to what is observed with MLG (Table 3). In terms of binding energies, the loss is localized on the side chain of Lys 2, that can mediate van der Waals interactions through its aliphatic chain but has an unfavorable electrostatic contribution due to the absence of stabilisation of the ammonium group (Figure S2 of Supp. Mat.).

**Table 3:** Influence of the single point alanine mutations on the lifetime of hydrogen bonds in hNaa50p.

Mutation	H112_O_	Y139_OH_	W	M75_O_	Y31_OH_	Y138_OH_
	M1_N_	M1_O_	M1_N_	L2_N_	L2_O_	G3_N_
WT M**L**G	**91.1**	**99.0**	**91.6**	**83.3**	**97.5**	**64.9**
WT M**K**G	83.6	99.1	80.2	77.2	94.4	58.9
F27A M**L**G	81.7	98.5	55.2	76.6	97.8	68.2
P28A M**L**G	91.8	99.1	23.5	72.6	98.3	75.2
V29A M**L**G	91.3	98.4	53.4	74.4	92.9	65.4
Y31A M**L**G	85.3	99.0	76.6	65.4	0	50.2
F35A M**L**G	85.3	98.6	90.0	66.2	47.1	65.9
Y73A M**L**G	86.2	98.8	61.7	69.8	98.4	64.9
H112A M**L**G	88.3	96.7	64.5	68.9	80.5	17.5
Y139A M**L**G	89.7	0	60.1	71.2	96.7	41.3
I142A M**L**G	89.7	98.8	49.5	64.3	92.4	53.1

Wild type hNaa50p is referred to as WT. Substrates are MLG peptides, with only one exception (MKG).

#### Ligand-enzyme interactions in hNaa50p/AcCoA/EEE

We then simulated hNaa50p with a substrate that is not efficiently acetylated by hNaa50p. The EEE peptide was thus modeled in the hNaa50p structure using the structure of MLGP as a template and MD simulations were then run following the same strategy as for the MLG-hNaa50p complex. Note that this is equivalent to “enforcing” EEE in the best possible position to interact with hNaa50p and following the interaction mode of MLGP. Obviously this is a rather artificial complex but we anticipated that these simulations would reveal which interactions are particularly unfavorable in this scenario. As expected, the presence of three negatively charged residues in the peptide modifies the stability of the complex. In particular, the presence of a glutamic acid at the N-terminal position yields an unfavorable contribution to the binding energy as there is no partner to accommodate the negative charge of its side chain (Figure 2B and 3). As a result, Glu1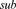 tends to move away from its starting position and the distance between N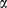 and the carbonyl group of the AcCoA is higher on average and less stable (5.2±2.2 Å with EEE as compared to 3.3±0.2 Å with MLG). This indicates that the presence of a glutamic acid as the first amino acid is not compatible with the ‘canonical’ anchoring of the N-terminal part of the peptide observed in the *3TFY* crystal structure and in the MLG-hNaa50p simulation. It follows that the acetylation can not take place. Interestingly the hydrogen bonds between the three tyrosines (31, 138 and 139) and the peptide backbone are still present but significantly weakened compared to MLG. Even for a peptide that is probably not able to bind to the enzyme, the three hydrogen bonds are possible and their energetic contributions are not unfavorable. This is also valid for the interaction of the N-Glu1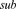 with His112. Side chains of the EEE substrate can mediate hydrogen bonds, but none of these interactions helps keeping the substrate close to the co-factor (see Figure 2B). Glu2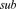 in particular can form extensive interactions with Arg62, but this interaction, despite participating in the anchoring of the peptide, might also impact the position of the N-terminal part by pulling it away from the co-factor. Glu3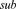 and Glu1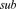 form hydrogen bonds with R141 but to a lesser extent.

The presence of the hydrogen bonds between tyrosines 31, 138 and 139 and the backbone of the EEE peptide, a highly unlikely substrate, shows the robustness of the three tyrosines as anchors for the peptide backbone. It reinforces the idea that they play a limited role in the ligand specificity of hNaa50p.

#### Effect of peptide binding on hNaa50p dynamics

**Figure 4:**
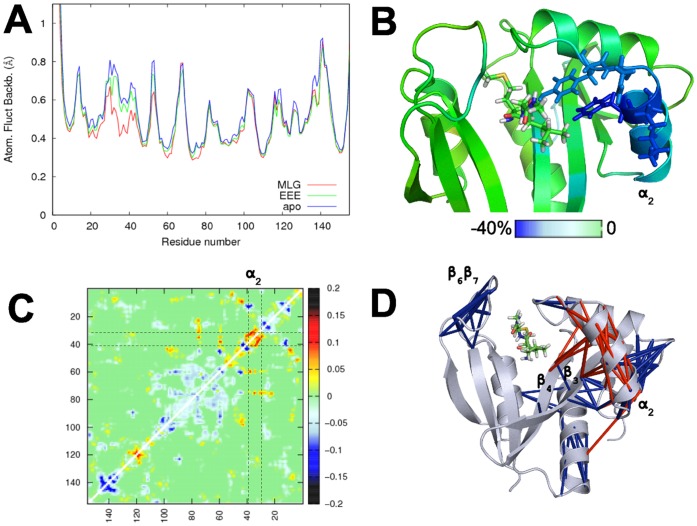
Effect of substrate binding on hNaa50p dynamics. A : Average atomic fluctuations in the simulations with MLG (red), EEE (green) and without substrate (blue). B: Changes in the backbone atomic fluctuations in hNaa50p resulting from MLG peptide binding. Amino acids of the protein are shown in sticks if their fluctuations are decreased by more than 30% compared to the *apo* simulation. C: Map of the differences of correlation between simulations of hNaa50p-MLG and hNaa50p-apo. Red dots indicate an increase in correlation, while blue dots indicate a decrease. Variations of anti-correlation are not represented on this map. D: Representation of the greatest gains (red) and loss (blue) of correlation between hNaa50p-MLG and hNaa50p-apo by sticks linking the corresponding amino acid pairs. Only the differences greater than 0.1 in absolute value are shown on this figure.

It is not unusual that ligand binding affects enzyme dynamics; changes can be local, i.e. restricted to the ligand binding and catalytic sites, or spread to other regions of the enzyme. In order to investigate the dynamics of hNaa50p as a function of peptide substrates binding, we simulated hNaa50p without peptide, (with only AcCoA bound, referred to as *apo* hNaa50p in what follows). We then compared the atomic fluctuations (RMSF) of the *apo* form with the simulations of hNaa50p with MLG and EEE peptides (Figure 4A). The insertion of both peptides reduces the fluctuations in the binding site, but the stabilisation is clearly stronger with the MLG peptide. Indeed, the presence of the EEE peptide results in lower fluctuations only for the residues directly participating in the stabilisation of the peptide, especially tyrosines Tyr31, Tyr138 and Tyr139. With the MLG peptide, however, the atomic fluctuations are decreased also at sites further away from the substrate and in particular in helix 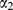. The largest stabilization occurs at the C-terminal end of helix 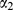, with clearly reduced fluctuations of residues Tyr31 to Phe35 (see Figure 4B). Note that data shown on Figure 4 correspond to averages over two or five simulations, the detailed atomic fluctuations (provided in Figure S3 of Supp. Mat.) lead to the same conclusions.

Next we performed an analysis of the quasi-harmonic modes obtained from trajectories of hNaa50p with and without MLG bound, and compared correlation of movements in the two systems. The correlation maps (shown in Figure S4 of Supp. Mat.) reveal differences that are best viewed on the difference map (Figure 4C). Pairs of amino acids undergoing the largest correlation changes from the apo to the holo form are represented on the structure of hNaa50p (Fig.4D) where we draw a stick between each pair. The sticks thus correspond to blue and red regions on the difference map. The most significant gains of correlation are localized on amino acids forming helix 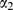, with an increase of the intra-helix correlation, but also increased correlation between this helix and strands 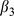 and 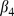. Several regions of the protein undergo a decrease of their correlations. The only one close to the catalytic site is loop 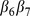 which plays a role in substrate interaction through Tyr138 and Tyr139, essentially.

The strongest effect of substrate binding on hNaa50p structure is thus localized on helix 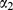. This effect is only observed with the substrate (MLG) that is experimentally known to be acetylated but not with EEE.

### Effects of Point Mutations on hNaa50p/MLG

**Figure 5:**
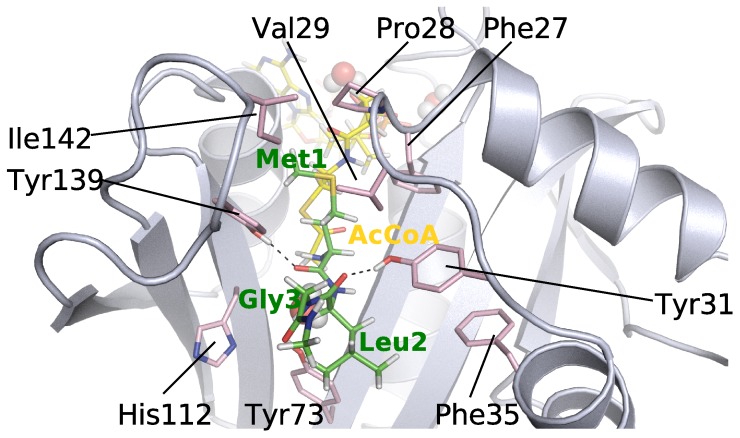
Position of alanine point-mutations in the hNaa50p structure, bound to the MLG peptide (green) and Ac-Coa (yellow). The peptide, co-factor and mutated residues are represented in sticks.

Single point mutations of amino acids Phe27, Pro28, Val29, Tyr31, Phe35, Tyr73, His112, Tyr139 and Ile142 into alanine result in a large decrease (at least ten fold, compared to the wild-type form, for all except Ile142) of the catalytic efficiency of the enzyme [15]. These amino acids are all located in or close to the ligand binding site (see Figure 5). Both the X-ray structure and our simulations of hNaa50p/MLG show that Tyr31 and Tyr139 interact through long-lasting hydrogen bonds with the backbone of Leu2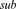 and Met1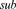 of the substrate, respectively. According to our simulations the other mutated amino acids interact with the peptide essentially through van der Waals interactions. Their contribution to the binding energies are about −1 kcal/mol (Val29) or less (Phe27, Pro28, Phe35, His112, Ile142, 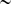 −0.5 kcal/mol) (Cf. Figure 3B).

We ran MD simulations of each of the hNaa50p mutants listed above, bound to AcCoA and MLG, and performed the same analysis as for the wild type enzyme (free energy decomposition, inventory of hydrogen bonds and changes in atomic fluctuations). We observe that the mutations have most of the time a local effect on the complex; the mutated amino acid is frequently the only one exhibiting a reduced contribution to the binding energy (Figure S5, Supp.Mat.) and each mutation at worse perturbs or disrupts only one hydrogen bond (Table 3). The mutations of Tyr31 and Tyr139 have the expected effects; Y31A affects the binding energy of Leu2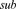 (backbone) which drops from −4 to almost 0 kcal/mol, while Y139A clearly affects the stability of Met1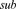 backbone-mediated interactions.

We identify a number of amino acids which appear to influence the position of their neighbors and by extension the overall structure of the **peptide** binding site. For example the backbone of His112 (also proposed to play a catalytic role along with Tyr73 [15]) forms stable hydrogen bonds with the peptide, both directly or bridged by a water molecule. The former remains unchanged compared to WT and the latter interaction is slightly affected by the H112A mutation. On the other hand the H112A mutation affects the Tyr138-Gly3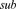 hydrogen bond, which has a reduced lifetime compared to simulations of the wild type (Table 3) and a reduced energetic contribution for the tyrosine. Interestingly it does not affect the binding energy of Gly3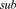. His112 thus seems to play a role in the orientation of the Tyr138 side chain. Tyr73 (also proposed to play a catalytic role along with His112 [15]) mediates moderate interactions with the peptide, but as His112, its mutation into an alanine affects the stability of the crystallographic water molecule which is bridging hydrogen bonds between Met1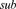 and His112 backbones. Finally exchanging Phe35 by an alanine reduces the lifetime of the hydrogen bond between the side chain of neighbouring Tyr31 and the Leu2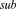 backbone, the latter yielding as a consequence a reduced contribution to the binding energy.

Residues Ile142, Phe27, Pro28 and Val29 are localized in the vicinity of Met1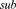 from the peptide. Their mutation results in a moderate energetic destabilization of Met1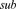 backbone-mediated interactions, illustrated by a decreased lifetime of the water-bridged hydrogen bond (Table 3). Moreover, in the case of F27A, the distance from substrate to AcCoA increases significantly (see Table 2). The moderate structural and energetic consequences of their mutation into alanine indicate that these bulky residues mainly participate in orienting Met1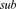 side chain.

**Figure 6:**
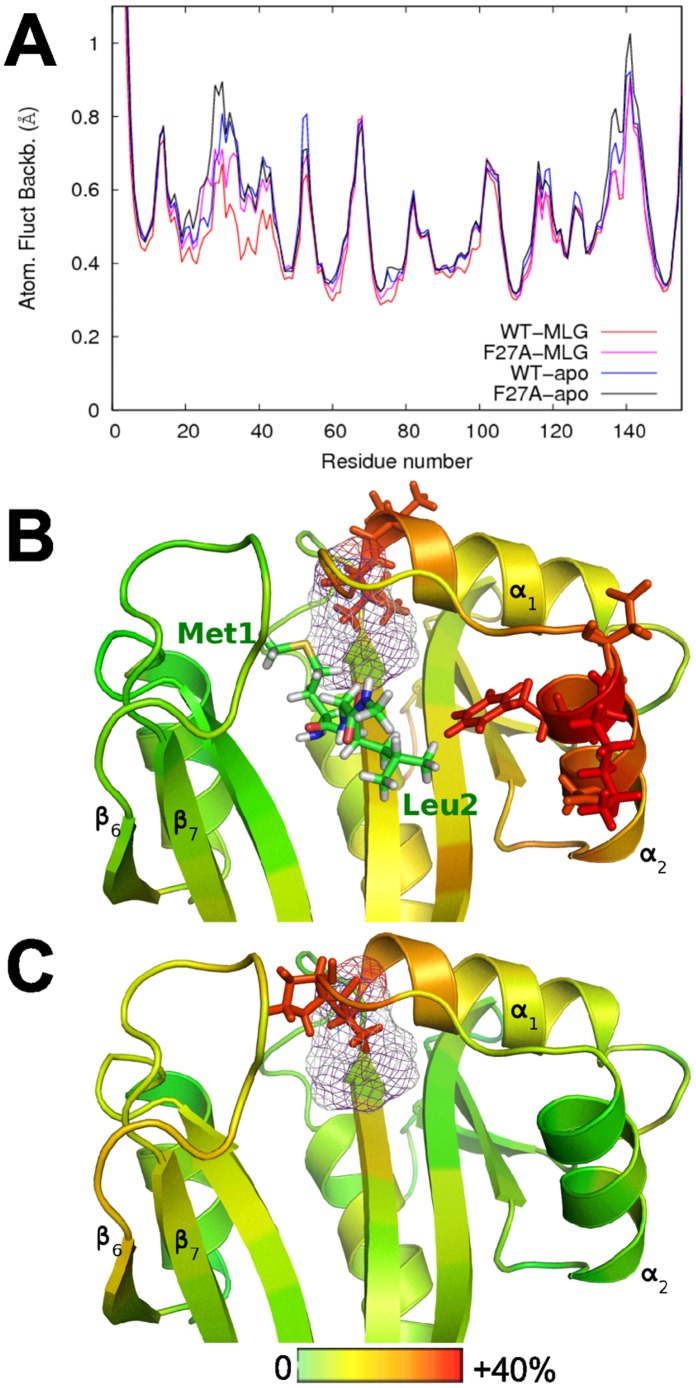
Effect of F27A point-mutation on hNaa50p dynamics. A: comparison of the average atomic fluctuations in simulations of the wild type and F27A mutant, with or without MLG substrate bound. B, C: Changes in backbone atomic fluctuations resulting from the F27A mutation, in presence or in absence of MLG substrate, respectively. Amino acids of the protein are shown in sticks if the difference of fluctuations exceeds 30%. Red colored regions become more flexible. The scale goes from 0 (no change, green) to 40% (red).

Analyses of the atomic fluctuations of complexes with alanine point-mutations (data given as Figure S6 of Supp. Mat.) reveal that some mutants induce a remote effect on the dynamics of the enzyme. We naturally expect an increase of the fluctuations near the mutation point due to the unfavorable effect on packing caused by the replacement of more voluminous amino acids by a smaller alanine side chain, which we do observe. In addition, mutation of F27, H112, I142 (all located in the vicinity of Met1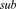), and to a lesser extent Y73, induce increased atomic fluctuations in the 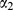 helix of substrate-bound complexes. We know that this region in the WT enzyme is stabilized by the binding of the MLG peptide (Figure 4B). These mutants thus seem to abolish at least partly the effect of the substrate on the 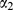 helix. This is illustrated with the example of F27A in Figure 6.

### Similarities between Catalytic Domains of Human NATs

#### Sequence conservation among catalytic domains of humans NATs

**Figure 7:**
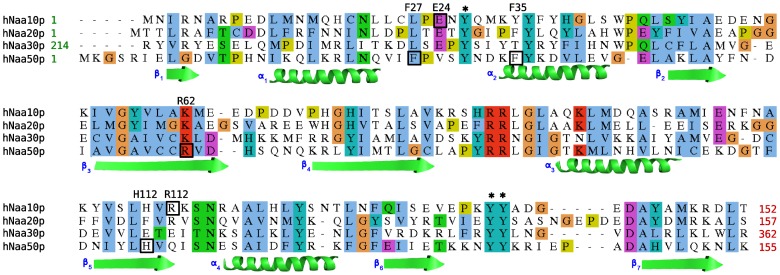
Sequence alignments of three catalytic domains of human NATs. The three black stars indicate the position of the tyrosines (31, 138 and 139) of hNaa50p. Residues of hNaa10p and hNaa50p identified by free energy decomposition are highlighted by black boxes and their name. We also represent secondary structure elements of the hNaa50p structure (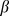-strands and helices).

The sequences of the different catalytic domains of human NATs share a low degree of sequence identity and hNaa40p has several sequence stretches that hNaa10p (NatA), hNaa20p (NatB), hNaa30p (NatC) and hNaa50p (NatE) do not share [16]. This makes any sequence alignment and subsequent structure prediction challenging. We thus focus here on these four catalytic domains (10p, 20p, 30p and 50p). The alignment of the four sequences is shown on Figure 7. The sequence identity with hNaa50p is *24*, *21* and *29%* for hNaa10p (NatA), hNaa20p (NatB) and hNaa30p (NatC), respectively. Tyrosines 31, 138 and 139 are conserved in all domains even though they have different substrate specificities. This suggests that the specificity is not attained using these three residues, but rather other amino acids of the enzymes. This is in line with our observation in the simulations of hNaa50p with different peptides.

**Figure 8:**
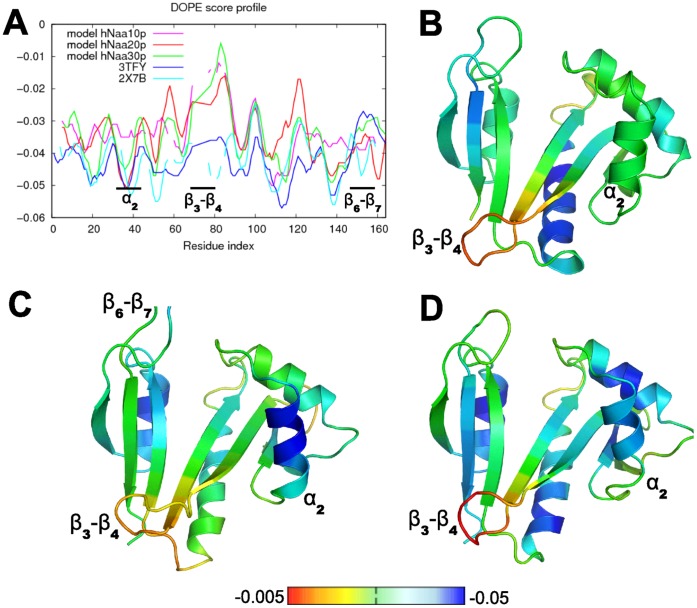
Evaluation of models of hNaa10p, hNaa20p and hNaa30p. A: DOPE score profiles for the structural models and the templates *3TFY* and *2X7B*. B, C, D: DOPE score profiles reported on the structures of hNaa10p, hNaa20p and hNaa30p, respectively, using a color gradient. Blue regions correspond to the lowest values (most reliable modelling), and red regions to the highest values.

We built models for the structures of hNaa10p, hNaa20p and hNaa30p. The model of hNaa10p was built using the structure of an archeal Naa10p (ARD from *Sulfolobus Solfataricus*, PDBID *2X7B*), as the human Naa10p shares a much higher sequence identity with the archeal Naa10p (36%, Cf Figure S7 of Supp. Mat.) than with the human Naa50p (24%). The models of hNaa20p and hNaa30p were built using the structure of hNaa50p (PDBID *3TFY*) since using the archeal protein would not significantly improve the sequence identity with the template, and thereby not lead to better models. The models were evaluated using a statistical potential (Discrete Optimized Protein Energy, DOPE [17]) which value is plotted for each model by amino acid residue on Figure 8. With the exception of the 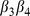 loop that has a higher DOPE values due to sequence insertions (but still lower than zero), the overall structures of the enzymes have DOPE scores indicating that the models are of reliable quality.

Because of the 34% sequence identity between human and archeal proteins, we regard the model of hNaa10p as the most reliable of the three and have performed MD simulations of this domain complexed with either the EEE or the MLG peptides. We subsequently performed the same analysis as for the complexes of hNaa50p to investigate the structural and dynamical basis of substrate specificity.

#### MD simulations of hNaa10p/EEE and hNaa10p/MLG

**Figure 9:**
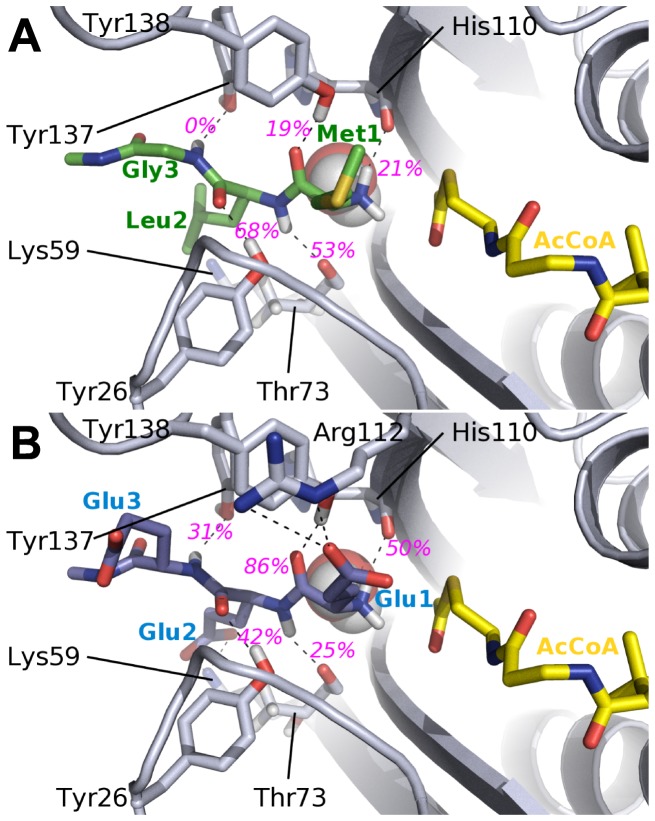
Representation of the substrate binding site of MLG and EEE peptides in hNaa10p. The peptides (MLG in green, EEE in blue), the side chains of amino acids (in grey) interacting with them, as well as the co-factor AcCoA (in yellow) are represented with sticks. The crystallographic water W is represented with spheres. The average lifetimes of the hydrogen bonds (dashed lines) involving peptide’s backbone during the last 6 ns of simulation are labelled in magenta.

**Figure 10:**
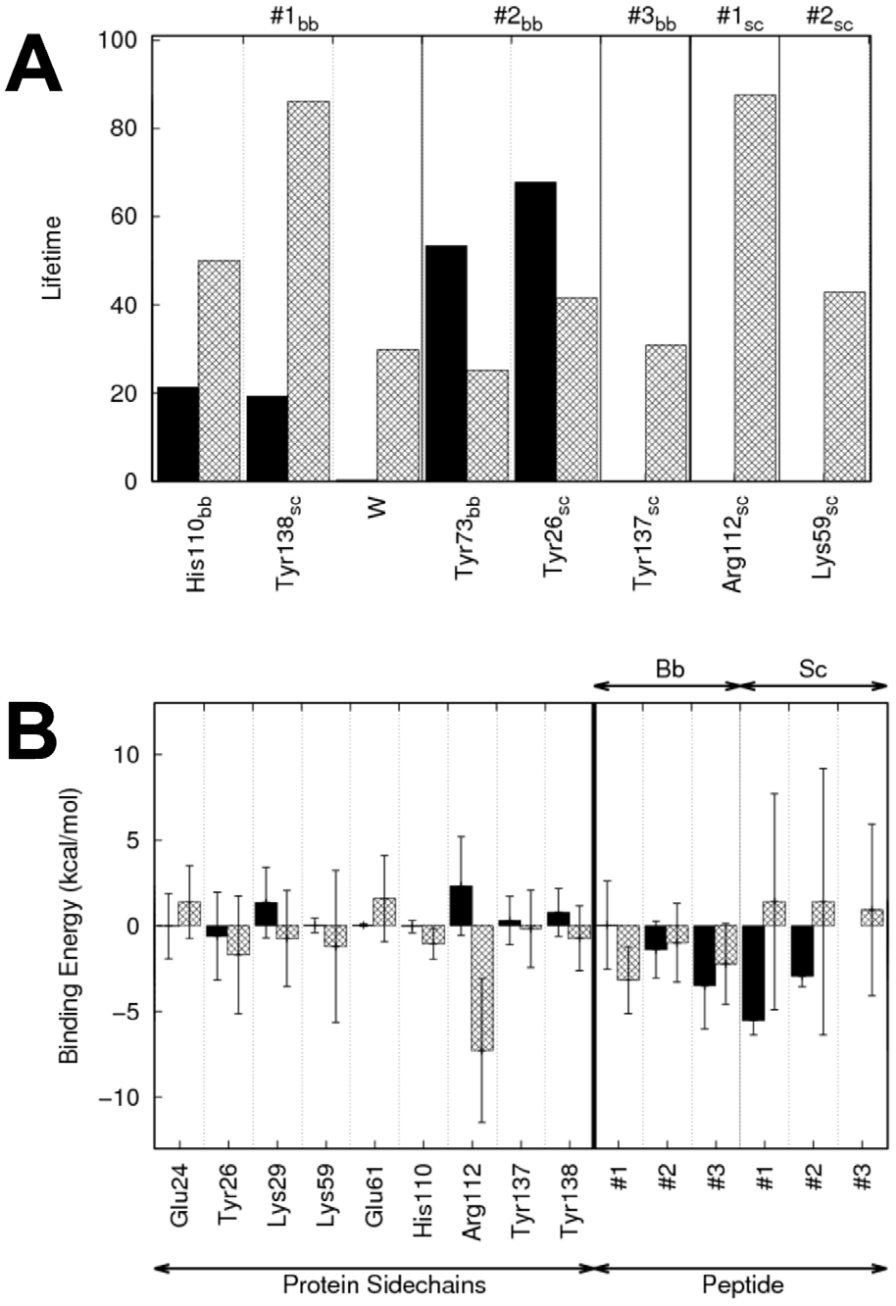
Hydrogen bonds lifetimes and contributions to the free energy of binding in hNaa10p. A: Average hydrogen bonds lifetimes during the last 6 ns of the simulations of hNaa50p with MLG (black bars) and EEE (grey) peptides. “bb” and “sc” correspond to backbone or side chain of the corresponding residue, respectively. #1, #2 and #3 are the peptide residue numbers. B: Amino acid residues of hNaa10p/MLG (black) and hNaa10p/EEE (grey) with the highest contribution to the free energy of binding (MM/PBSA). The contributions are divided between backbone (bb) and side chains (sc) contributions.

The hydrogen bond pattern of hNaa10p with EEE shares a number of similarities with the complex between hNaa50p and MLG. Figure 9A shows the hydrogen bonding network between hNaa10p and the EEE peptide. The three conserved tyrosines interact significantly with the peptide backbone although their lifetime is lower than what we observed for hNaa50p/MLG (see Figure 2A). The number and strength of these hydrogen bonds indicate clearly stronger hydrogen bonds between hNaa10p and EEE as compared to MLG in agreement with activity assays [12]. In particular, the hydrogen bonds stabilizing Glu1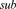 in EEE have much higher lifetimes than the ones with Met1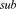 in MLG (Cf. Figure 9). Moreover the presence of Arg112 favors a hydrogen bond acceptor group at the N-terminal position. We indeed observe a strong hydrogen bond between Glu1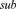 and Arg112 (88% lifetime), and the side chain of Arg112 has the highest contribution (ca. −7 kcal/mol) to the binding energy of the complex (see Figure 10B, grey bars). Such an interaction is not possible in hNaa50p as there is no positively charged amino acid in the neigborhood of the active site where the N-terminus of the peptide is located. Glu2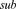 can also form hydrogen bonds with Lys59 (Cf Figure 9B), an interaction that appears equivalent to the Glu2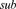-Arg62 hydrogen bond in hNaa50p/EEE, but with a lower lifetime (43% as compared to 95%, respectively). Still the most important stabilisation of the EEE peptide in hNaa10p is at the N-terminal position. The distance between the N-terminus and the acetyl group of AcCoA is on average slightly higher than for hNaa50p/MLG but it also fluctuates more. This can possibly be explained by the resolution of our model. Indeed homology models built with a low sequence identity (here 36%) intrinsically have a lower resolution than a X-ray structure.

In the hNaa10p/MLG complex, we observe unusually weak binding energies for protein amino acids and short lifetimes for the hydrogen bonds (black bars in Figures 10A and 10B, respectively), if they exist. This underlies the fact that this complex is particularly unstable during the MD simulations. While all five simulations of hNaa10p/EEE converge to distances between N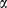 and the carbonyl group of the AcCoA of around 4 Å we observe much higher distances during the simulations of hNaa10p/MLG, and especially so towards the end (up to 10 Å, Cf. Figure S8 of Supp. Mat.). The average over the last 3 ns of the simulations is 5.9 Å (±1.9 Å), which is the same order of magnitude as in the hNaa50p/EEE complex (5.2±2.2 Å). Both values are high compared to the values of the true enzyme-substrate complexes hNaa10p/EEE (4.2±0.6 Å) and hNaa50p/MLG (3.3±0.2 Å).

#### Structural basis for substrate specificity in hNaa20p and hNaa30p

The simulations of hNaa50p described above (with three different substrates and nine mutants) reveal the sequence and structural determinants for ligand recognition for an archetypal human N-terminal acetyltransferases. Simulations of the homology model of hNaa10p show that the recognition patterns are similar (similar regions of the structure determine the specificity). Thus, based on this knowledge, and although the resolution of the models of hNaa20p and hNaa30p is limited due to the low sequence identity with the template used (hNaa50p), we are able to propose a model for the substrate interaction in the latter enzymes.

**Figure 11:**
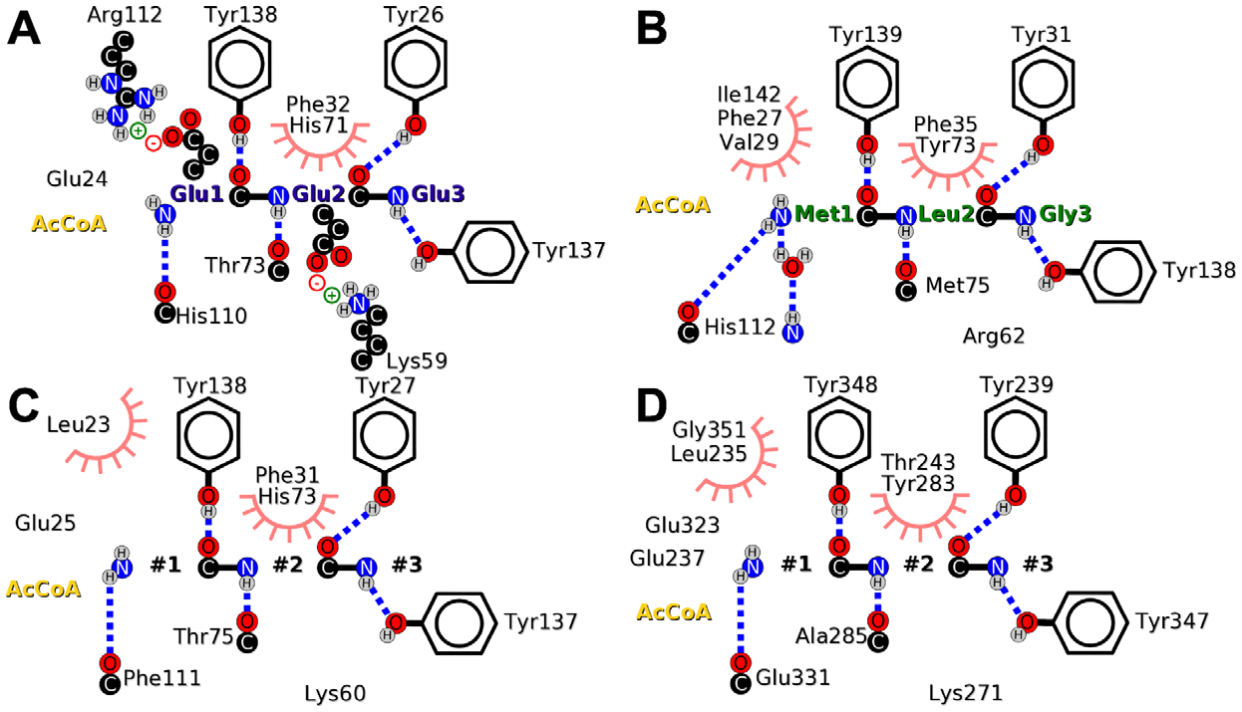
Models of the substrate binding sites of hNaa10p (A), hNaa50p (B), hNaa20p (C) and hNaa30p (D). Hydrogen bonds are shown in dashed blue lines, while residues involved in hydrophobic contacts with the substrate are represented with a pink half-circle.

The amino acids observed in the substrate binding sites are represented on Figure 11. A decisive difference between hNaa10p and hNaa50p, as mentioned above, is the presence of Arg112 in hNaa10p which can interact with hydrogen bond acceptors such as Asp or Glu as the first amino acid of the peptide substrate. hNaa50p instead has a rather hydrophobic pocket. In hNaa20p and hNaa30p, this pocket is less hydrophobic according to our models. It still seems to be able to accomodate non polar residues, in agreement with experimental data that shows that it acetylates N-terminal methionines [12]. In both enzymes, the pocket for S1’ is in addition lined by one and two glutamic acids for hNaa20p and hNaa30p, respectively. This would make it unlikely that hNaa30p in particular can acetylate substrates with an acidic N-terminal residue. On the contrary our model suggests that it can acetylate N-terminal amino acids containing hydrogen bond donors such as Ser, Thr, Lys or Arg. Unlike in hNaa30p, Arg112 is conserved in hNaa20p but it seems to be more prone to interact with the extended 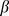
6–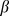
7 loop that contains additional aspartic and glutamic acids. That would limit its ability to interact with acidic S1’. The P2’ site of hNaa20p is very similar to its counterpart in hNaa10p: Tyr26, Phe32, Lys59, His71 and Thr73 are conserved. Its ability to acetylate substrates with hydrophilic (Asn, Gln) or acidic (Asp, Glu) amino acids in S2’ [18] can be explained by the presence of Lys60. In hNaa30p, compared to hNaa50p, Phe35 becomes a threonine (Thr283) and Met75 is replaced by an alanine (Ala285). The S2’ site of hNaa30p is thus able to accommodate larger amino acid side chains than hNaa50p and that explains its ability to acetylate peptides with a Trp or Phe as P2’ [19]. In general the ligand binding site of hNaa20p is closer to the one in hNaa10p than to hNaa50p.

### Conclusion

We have set up a computational protocol to build and simulate complexes between hNaa50p and several peptides. We validated this approach by comparing our results with the X-ray structure of hNaa50p bound to the MLG peptide. In particular, the interaction between substrate and enzyme are well reproduced and the distance between the AcCoA and the N-terminus is compatible with the reaction mechanism. We could thus use the same computational strategy to investigate other complexes of hNaa50p. Simulations show that the enzymes stabilize the tripeptide substrates with decreasing energetic contributions from the first substrate amino acid to the third. The hydrogen bonds between three conserved tyrosines (31, 138 and 139 in hNaa50p) and the peptide backbone are robust to sequence variation in substrate and are conserved between 4 different catalytic domains of human NATs, even though they have different substrate specificities. These tyrosines do not play a decisive role for substrate specificity but rather form a scaffold ready to interact with peptide backbones. The specificity is rather tuned by surrounding amino acids that are not conserved between the different NAT. Comparing the sequence and structure of hNaa50p and hNaa10p to sequences and structural models of hNaa20p and hNaa30p allows us to propose a map of the amino acids that seem important for the specificity (see Figure 11). The largest differences are observed in S1’; hNaa10p, with its Arg112 is able to stabilize P1’ acidic amino acids, while hNaa50p has a hydrophobic S1’ site. Though hNaa20p and hNaa30p are known to acetylate substrates with a methionine N-terminus, they appear to offer a favorable site for basic amino acids as well. hNaa30p has a larger binding site for S2’ than the other 3 enzymes thanks to the mutation of a conserved Phe to Thr, and the replacement of Thr(hNaa10p, hNaa20p) or Met(hNaa50p) to an alanine. All four S2’ sites are rather hydrophobic although they all have a basic amino acid (Lys or Arg) that can interact with acidic P2’ amino acids. Lys59 of hNaa10p interacts with Glu2 of the EEE peptide, and so does Arg62 of hNaa50p even though the interactions in the S1’ site are unfavorable. The conservation of this basic amino acid might be the sign of the versatility of S2’. We believe that the maps on Figure 11 will be useful for designing mutagenesis experiment to further investigate the substrate specificity *in vitro* of hNaa20p and hNaa30p. Such experiments would be useful since the structural data available to us at this date remains of lower quality for these two enzymes than for hNaa50p.

Simulations of hNaa50p and its mutants showed that the substrate binding site is dynamically correlated with helix 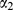. Binding of the MLG peptide strengthens the correlation between helix 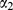 and the substrate binding site, and rigidifies the helix. Alanine mutations known to reduce the catalytic activity slightly affected the interactions with the substrate and that was enough to suppress the effect on the dynamics of helix 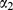. These results indicate that long-range communication might exist between the catalytic site and the 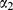 helix. Yet the actual existence of this communication path and its functional relevance remain to be demonstrated by, for example, catalytic data of mutants of amino acids belonging to the 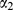 helix.

Our study focuses on the isolated catalytic subunits of NATs. It is important to keep in mind that their specificity might be modulated when they are involved in larger complexes. Until structural data on the complexes becomes available our study provides atomic level of details rationalizing substrate specificity of the catalytic domains and the basis for the design of specific inhibitors targeting the human NATs.

## Methods

### Homology Modeling

The homology model of hNaa10p was built with Modeller version 9.7 [20] using as template the Ard1 catalytic domain of *Sulfolobus Solfataricus* (PDBID : *2X7B*). Indeed hNaa10p has a much higher sequence identity with the archeal Naa10p (36%, Cf Figure S7 of Supp. Mat.) than with the human Naa50p (24%). The models of hNaa20p and hNaa30p were built using the structure of hNaa50p (PDBID *3TFY*). The sequence alignments were realized using Clustal W [21]. In each case, 150 models were generated and evaluated using the Discrete Optimized Protein Energy (DOPE) [17] potential, also referred to as DOPE score. The models with the lowest overall DOPE score were selected. DOPE values per amino acid were plotted to characterize potential low quality regions in the models. The 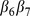 loop of hNaa20p was optimized using the *loopmodel* class in Modeller.

### Force Field Parameters for AcCoA

We developed new parameters for the thioester moiety of AcCoA Geometry optimization of ethyl acetate and its thioester equivalent were performed with Gaussian03 using HF/6-31G*, and Mulliken charge repartitions of both compounds were compared. These differences were then used to adapt the Charmm charges of ester groups to thioester. Parameters and charges for the nucleotide and phosphate part were copied from the fragment adenosine (ADE of Charmm27). For the rest of AcCoA, internal and van der Waals parameters as well as charges were either copied or derived by similarity from existing Charmm27 parameters. Using MD simulations of AcCoA complexed with hNaa50p, we checked that we could reproduce the interactions between AcCoA and the enzyme observed in the X-ray structure. The final parameters are given in Supp. Mat. (S9).

### Systems Preparation

As hNaa50p was crystallized with CoA instead of AcCoA, we used the structure of a GCN5 histone acetyltransferase bound to AcCoA as a template (PDBID *1Z4R*) to orient the acetyl group in hNaa50p. The mutations of side chains on the peptide were performed manually using PyMOL [22]. Docking of the peptide in hNaa10p model was achieved by superposition on the hNaa50p crystallographic structure.

The protonation states at the crystallisation pH of all histidines were determined using PROPKA [23]. This lead to the following result: in hNaa50p 4 histidines were protonated on N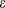 and 3 on N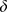, while for hNaa10p the repartition was 3 N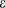 and 2 N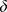. All other titratable groups were placed in their standard protonation states. Hydrogen atoms were constructed using the HBUILD module of the CHARMM program [24]. The terminal residues of the protein, as well as the C-terminal residue of the peptide, were constructed in their charged state (NH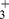 and COO^–^). For the N-terminal residue of the peptide, a neutral (NH_2_) amine group was used. Indeed, preliminary simulations of hNaa50p/MLG with a charged N-terminal peptide lead to a high instability of the complex (data not shown). The complexes were solvated in cubic boxes of TIP3P water [25] of 80Å, large enough to prevent any interaction between the complex and its images. Water overlapping the proteins, determined by a cutoff of 2.8Å was removed.

### Molecular Dynamics

The crystallographic structure of hNaa50p (PDBID : *3TFY*), as well as the homology model of hNaa10p, have been simulated with both EEE and MLG peptides. Molecular dynamics (MD) simulations were used to explore the conformationnal space around the starting structures. In order to increase conformational sampling around the starting structures, we chose to perform several simulations of each complex rather than a long trajectory. Multiple short simulations are indeed known to induce a better sampling than a single longer trajectory [26]. Thus, for each case, five trajectories of 10 ns length were ran, each using a different initial distribution of velocities.

The MD procedure used was as follows. Simulations were performed at a temperature of 300K using the NAMD program [27] and the CHARMM27 force field [25]. The SHAKE algorithm was used to constrain all bonds between a heavy atom and a hydrogen. Non-bonded interactions were truncated at a cutoff of 14Å, using a switch function for van der Waals, and a shift function for electrostatics [28]. The particle-mesh Ewald algorithm [29] was used to evaluate the long range electrostatic interactions. The system was subjected to an energy minimisation of 1000 steps using the conjugated gradient algorithm, followed by four heating steps to 10K, 100K, 200K and 300K, respectively. This was followed by a 150 ps equilibration phase during which velocities were reassigned every picosecond. The production phase consisted in 10 ns simulation in the NPT ensemble, with a timestep of 1fs.

In the case of the homology model of hNaa10p, harmonic distance restraints between hydrogen bond acceptor oxygens and donor hydrogens were introduced during the heating and equilibration procedures, and the equilibration phase was extended to 1 ns. The aim of these restraints was to stabilize the hydrogen bonds between the backbone of the docked peptide and the homology model. All restraints were removed during the production phase.

### Analysis

#### Free energy decomposition

A protocol based on the MM/PBSA method was used to obtain a semi-quantitative evaluation of the contribution of all amino-acids to the formation of the complex. In this approach (described in [30]), the free energy is expressed as the sum of terms of equation 1.
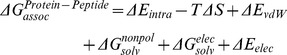
(1)


Two approximations have been introduced. First, due to the protocol based only on a trajectory of the complex, the change of internal energy upon the complex formation is neglected. Also, changes in conformational entropy are neglected as they have been shown to contribute to increasing quantitative agreement, but not change general trends [31], [32]. These approximations result in a semi-quantitative estimation of the binding energy, which can be decomposed into individual contributions of each amino-acid of the complex following the protocol presented in Lafont et al [30]. The solvent contribution to the electrostatics terms is calculated using the University of Houston Brownian Dynamics program (UHBD) [33] and the intermolecular electrostatics term is calculated using the partial charges in the CHARMM force field [25]. The van der Waals and non polar contributions are evaluated using the CHARMM program [28]. The non-polar contribution is taken proprotional to the change of Solvation Accessible Surface Area (SASA).

The protocol is based on the extraction of an ensemble of representative conformations from the MD simulation. It has indeed been observed that small structural changes can lead to significant variations in terms of energy in the MM/PBSA method [34]. As it is known that Coulomb energy reflects conformational changes, this energy is calculated for all conformations saved from the trajectory, that are then sorted and clustered into groups that are affected a weight given their population. For each cluster, the representative conformation is the one with the energy closest to the cluster average.

For a given complex, free energy decomposition is calculated on 25 representative conformations taken from ensembles of conformations that contain the last 2.5 ns of two or five simulations. Final free energy contributions and standard deviations are calculated as weighted averages of the representative conformations, the weight of a cluster corresponding to its population.

#### Other analyses

Hydrogen bonds, atomic fluctuations and quasi-harmonic analysis were performed on the last 6 ns of each trajectory, where systems had reached equilibrium (RMSD plots available in Figure 1, and in Figure S1 of Supp. Mat.). For each system, we present results that correspond to averages over two simulations (most of the time) or over five simulations (hNaa10p/hNaa50p in complex with EEE/MKG), that we call replicates. Detection of hydrogen bonds was achieved using a 2.4Å distance criterion between hydrogen and acceptor, and a 130° donor-hydrogen-acceptor angle criterion. Atomic fluctuations were calculated on 12 windows of 500ps each using the average structure for each respective window. Quasi-harmonic analyses were performed with CHARMM [28]. A mass-weighted reorientation was used to remove net translation and rigid-body rotation of the system. Cross-correlations were calculated by normalizing the covariance matrix.

## Supporting Information

Figure S1 **Backbone RMSD of hNaa50p during MD simulations.** The plots correspond to the simulations of the alanine mutants (F27A, P28A, V29A, Y31A, F35A, Y73A, H112A, Y139A and I142A) in complex with an MLG peptide, of a wild-type/MKG complex, and of the *apo* form of the F27A mutant. RMSD has been calculated after superposition of the trajectory on the starting structure. Red and green lines correspond to two different replicas.
(PDF)

Figure S2 **Contributions to the free energy of binding in hNaa50p.** Amino acid residues of hNaa50p/MLG (black) and hNaa50p/MKG (grey) with the highest contribution to the free energy of binding (MM/PBSA). The contributions are divided between backbone (bb) and side chains (sc) contributions.
(TIF)

Figure S3 **Detailed atomic fluctuations during the simulations of hNaa50p**. Data is provided for the 5 simulations of the enzyme in complex with MLG (red) and EEE (green), and the 2 simulations of the *apo* hNaa50p (blue).
(TIF)

Figure S4
**Correlation maps of hNaa50p** ***apo*** **(above diagonal) hNaa50p/MLG (below diagonal).** Correlations are shown in a yellow to red gradient, and anti-correlations in blue. The position of the 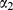 helix is highlighted by the dashed lines.(TIF)

Figure S5 **Free energy decompositions (expressed in kcal/mol) of alanine point-mutants of hNaa50p/MLG.** Black bars are used for the wild-type hNaa50p/MLG and white dashed bars for the mutant (F27A, P28A, V29A, Y31A, F35A, Y73A, H112A, Y139A, I142A).
(PDF)

Figure S6 **Change in backbone atomic fluctuations of hNaa50p/MLG resulting from alanine point-mutations.** Plots (left) compare the atomic fluctuations of the mutant (P28A, V29A, Y31A, F35A, Y73A, H112A, Y139A, I142A) and wild-type, while the relative difference is shown on the structure (right). Amino acids of the protein are shown in sticks if the difference of fluctuations exceeds 30%. The scale goes from −40% (blue : decreased flexibility) to +40% (red : increased flexibility).
(PDF)

Figure S7 **Sequence alignment between hNaa10p and the template from** ***Sulfolobus Solfataricus*** **(PDBid** ***2X7B*** **)**
(TIF)

Figure S8
**Distance (Å) between the N-terminal nitrogen of Met1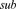 and the carbon atom of Ac-CoA carbonyl in hNaa10p complexed with EEE (top) and MLG (bottom).** In each case we represent the evolution of the distance for the five simulations of the corresponding complex.(TIF)

Figure S9 **CHARMM force field parameters for AcCoA.** Parameters provided in the CHARMM topology format.
(PDF)
