# Supplementary figures and images for: Specificity and Versatility of Substrate Binding Sites in Four Catalytic Domains of Human N-Terminal Acetyltransferases
Source: PLoS One. 2012 Dec 28;7(12):e52642. doi: 10.1371/journal.pone.0052642 (PMC3532069; doi:10.1371/journal.pone.0052642)

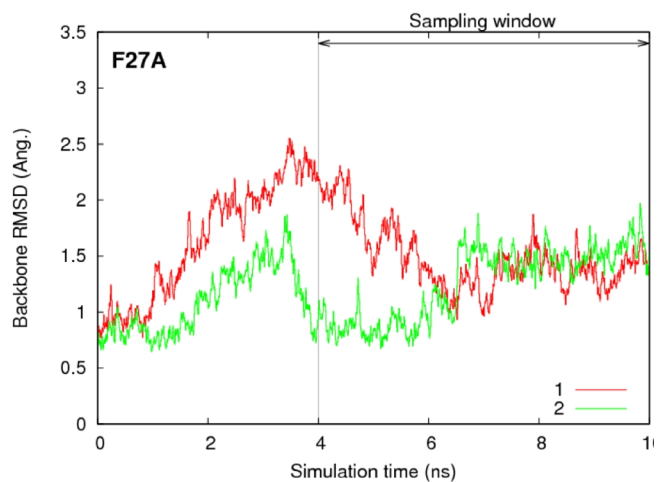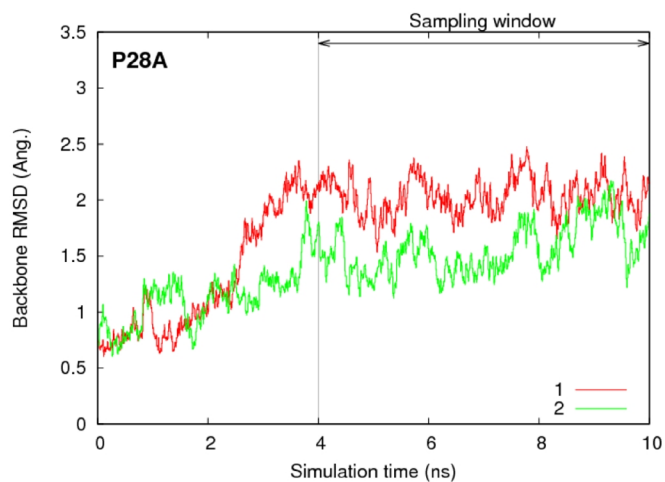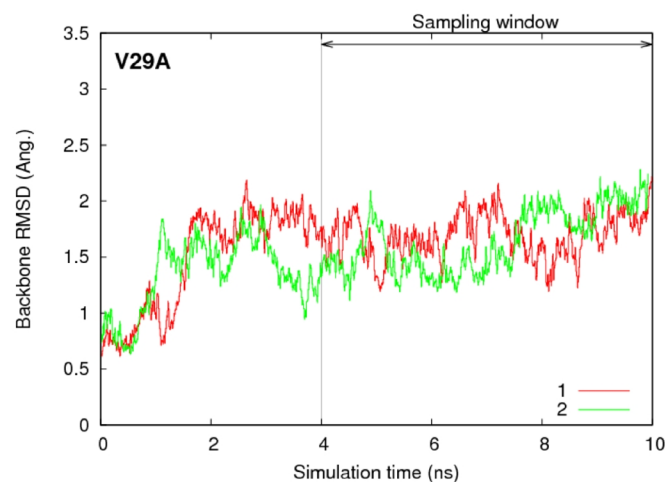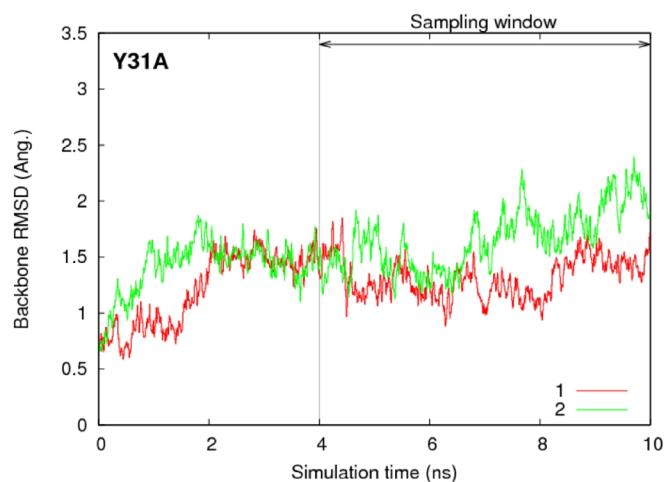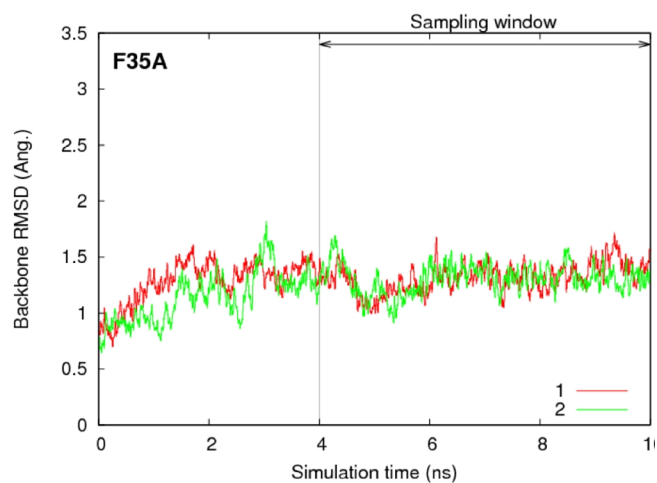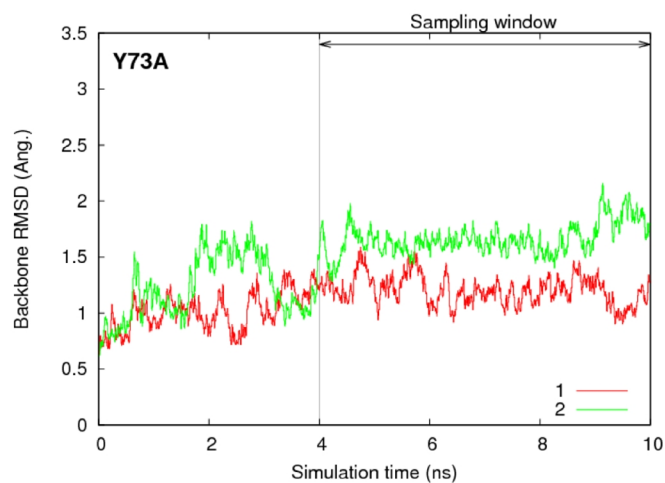

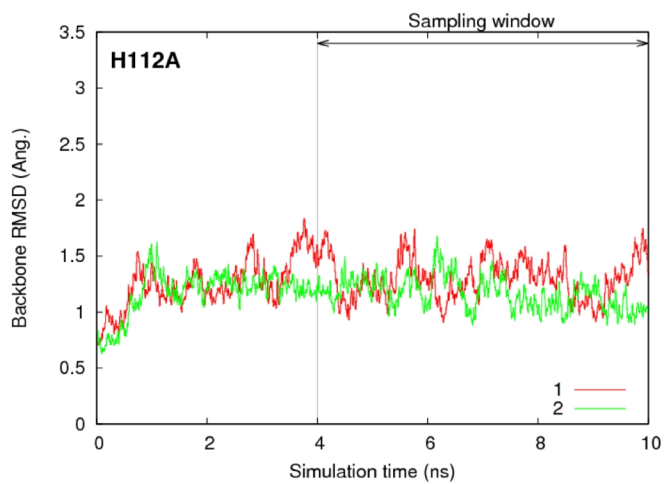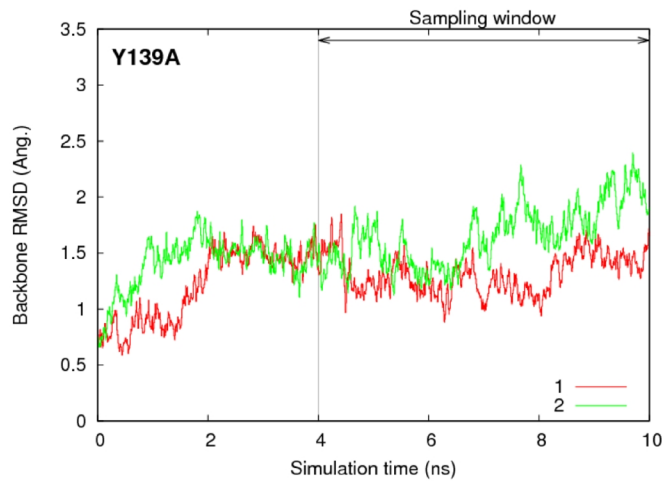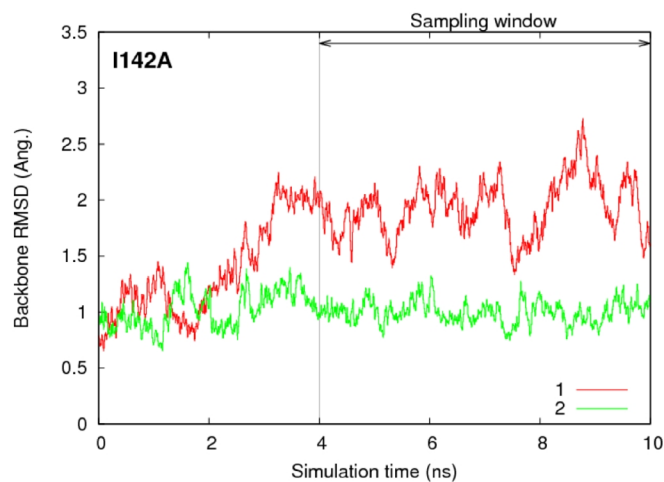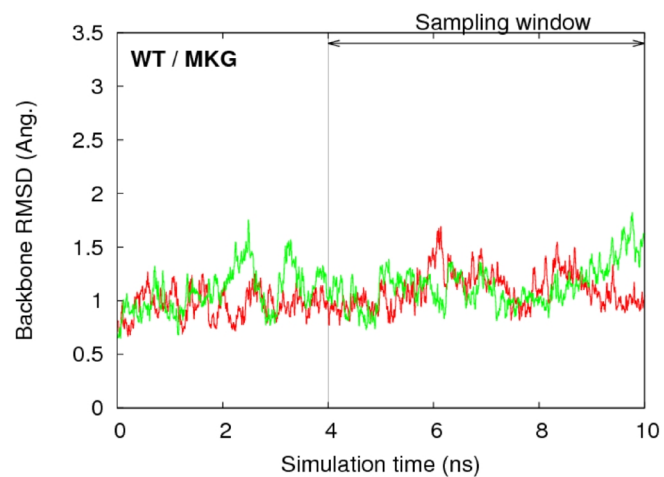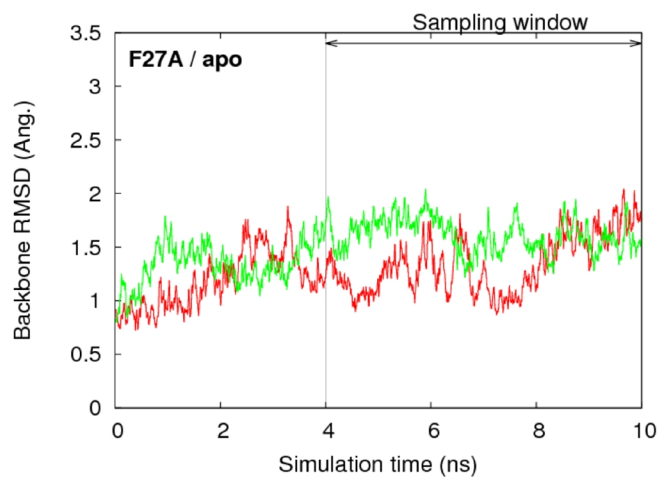

Supplement: Figure S1 — Backbone RMSD of hNaa50p during MD simulations. The plots correspond to the simulations of the alanine mutants (F27A, P28A, V29A, Y31A, F35A, Y73A, H112A, Y139A and I142A) in complex with an MLG peptide, of a wild-type/MKG complex, and of the apo form of the F27A mutant. RMSD has been calculated after superposition of the trajectory on the starting structure. Red and green lines correspond to two different replicas. (PDF) [file pone.0052642.s001.pdf]

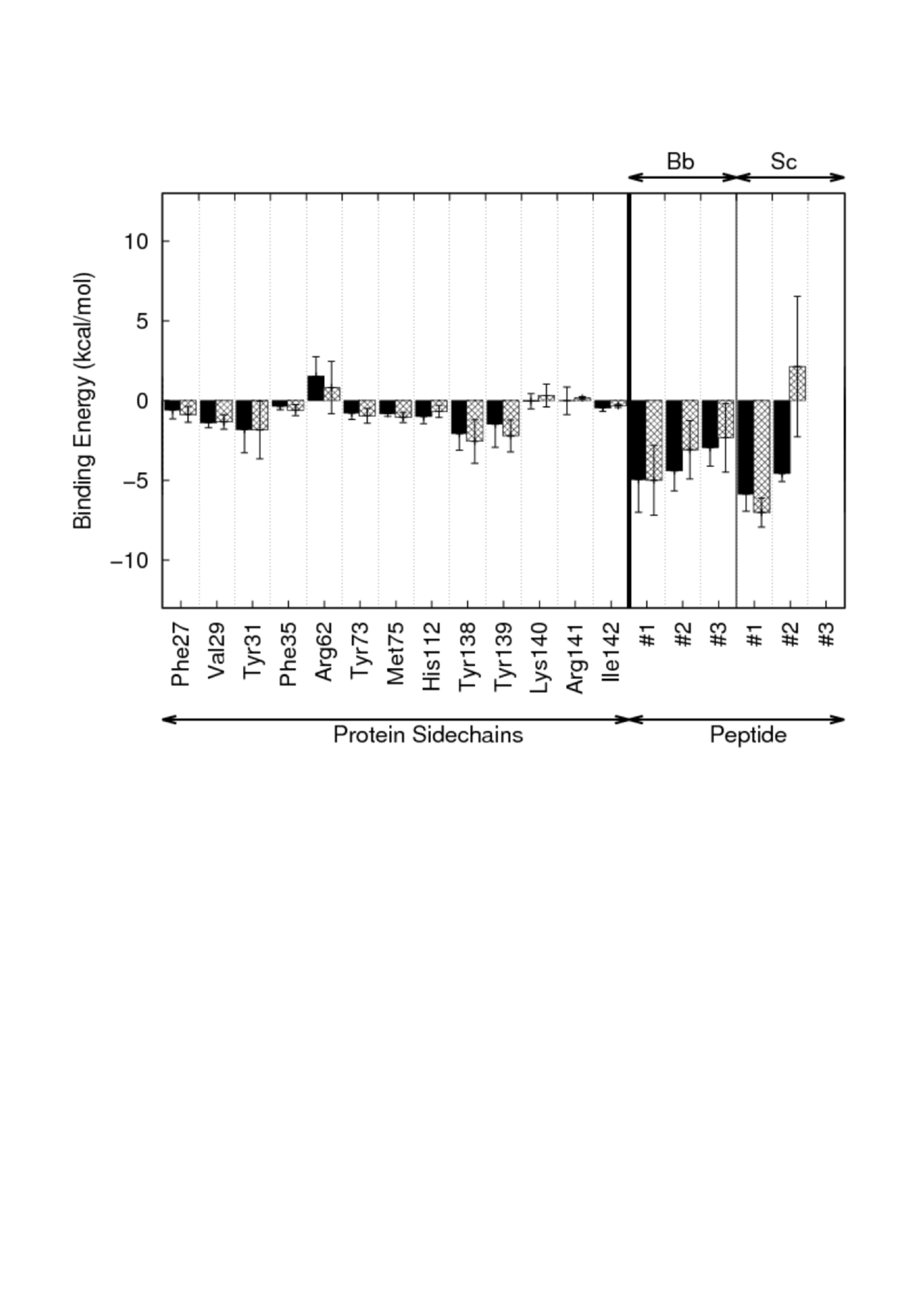

Supplement: Figure S2 — Contributions to the free energy of binding in hNaa50p. Amino acid residues of hNaa50p/MLG (black) and hNaa50p/MKG (grey) with the highest contribution to the free energy of binding (MM/PBSA). The contributions are divided between backbone (bb) and side chains (sc) contributions. (TIF) [file pone.0052642.s002.tif]

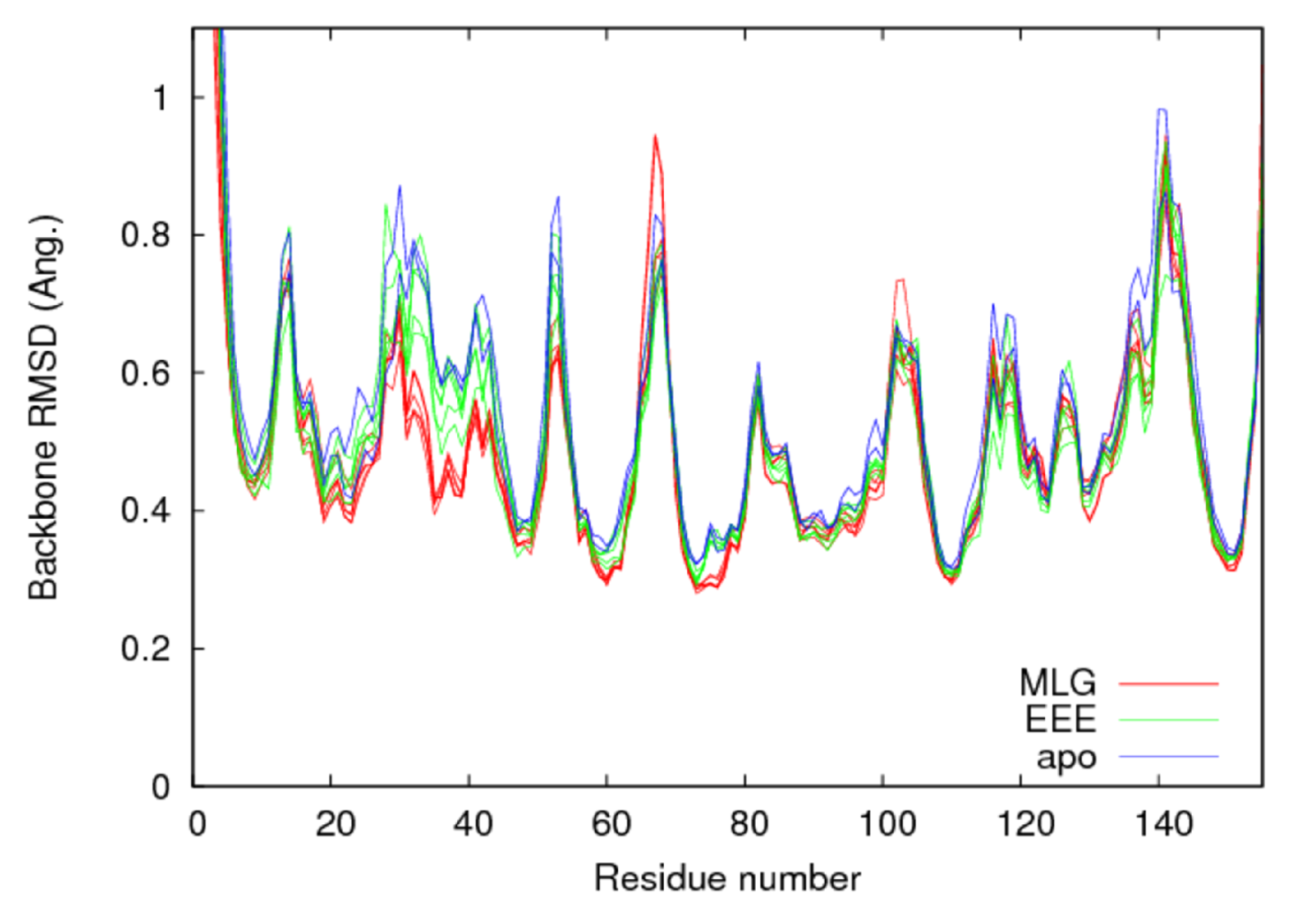

Supplement: Figure S3 — Detailed atomic fluctuations during the simulations of hNaa50p. Data is provided for the 5 simulations of the enzyme in complex with MLG (red) and EEE (green), and the 2 simulations of the apo hNaa50p (blue). (TIF) [file pone.0052642.s003.tif]

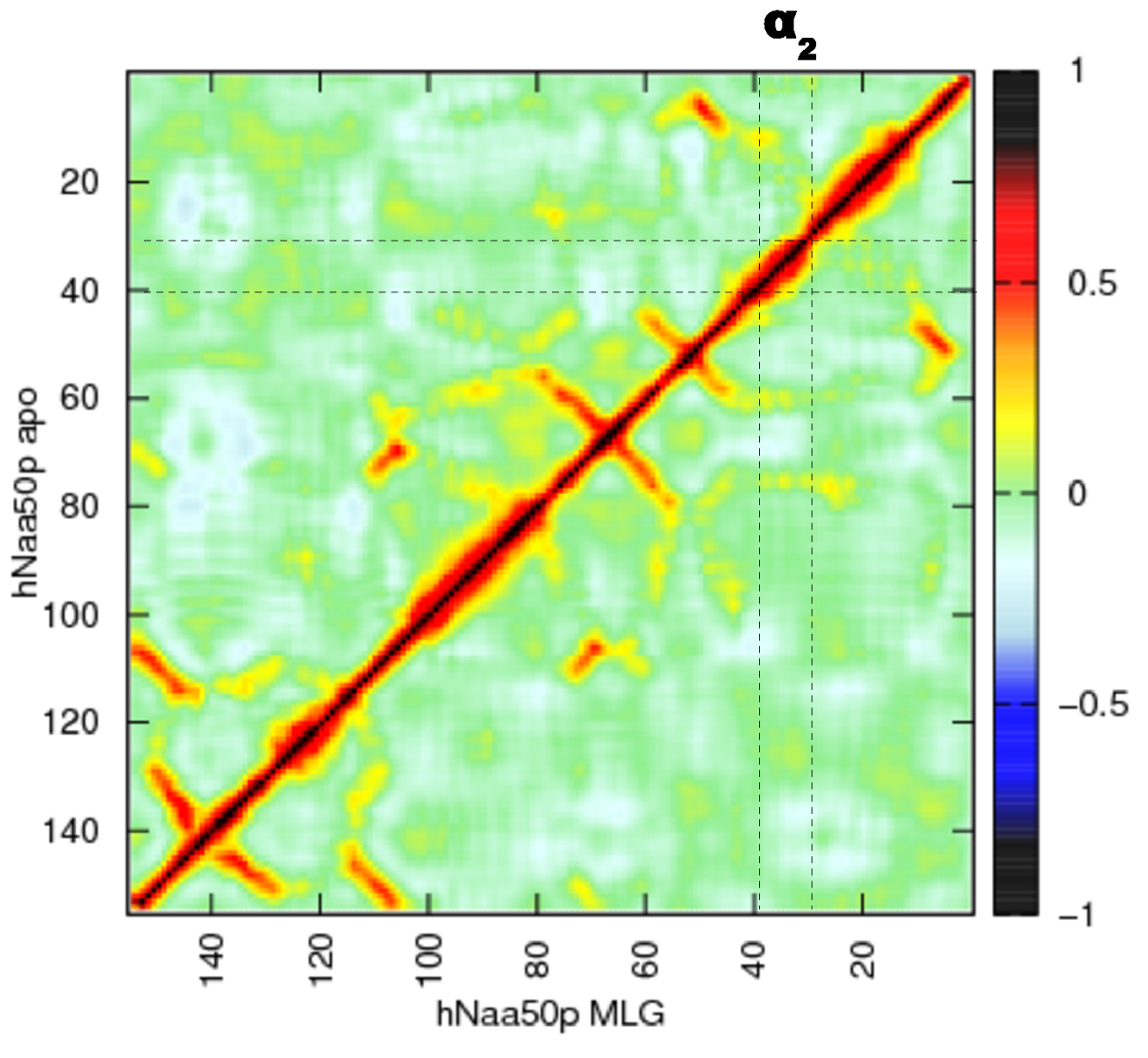

Supplement: Figure S4 — Correlation maps of hNaa50p apo (above diagonal) hNaa50p/MLG (below diagonal). Correlations are shown in a yellow to red gradient, and anti-correlations in blue. The position of the helix is highlighted by the dashed lines. (TIF) [file pone.0052642.s004.tif]

**F27A**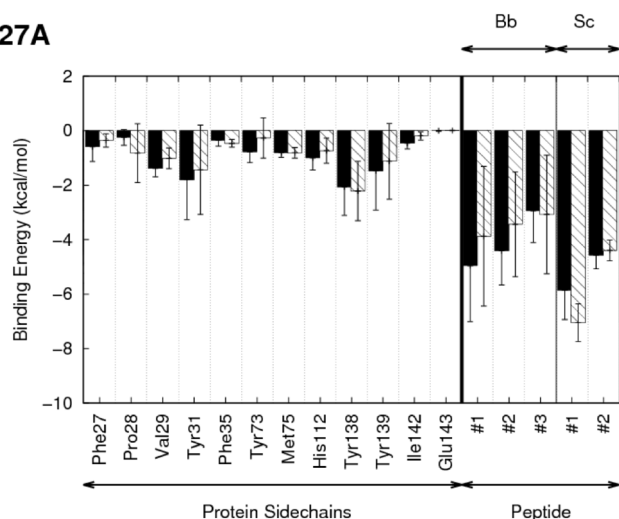**P28A**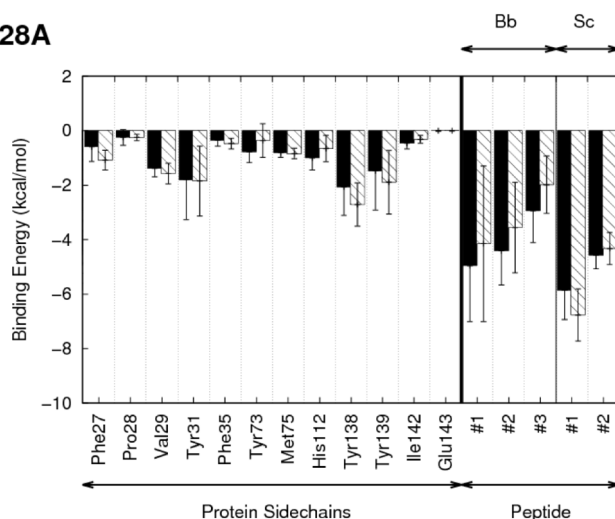**V29A**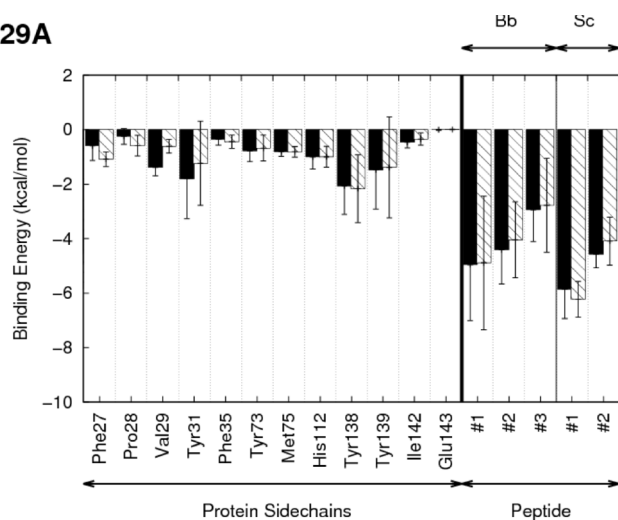**Y31A**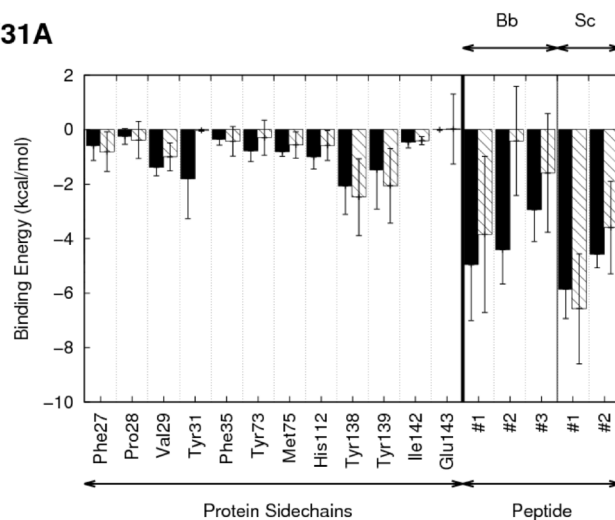**F35A**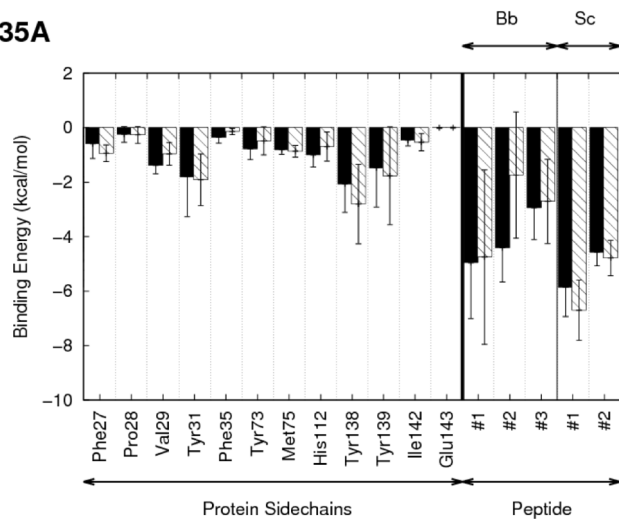**Y73A**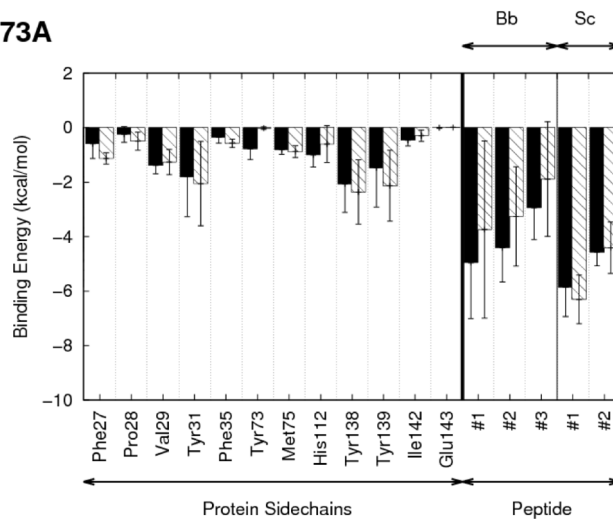

H112A

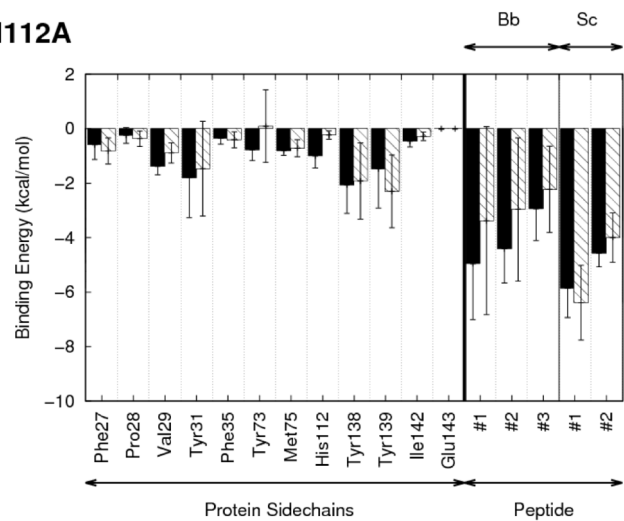

Y139A

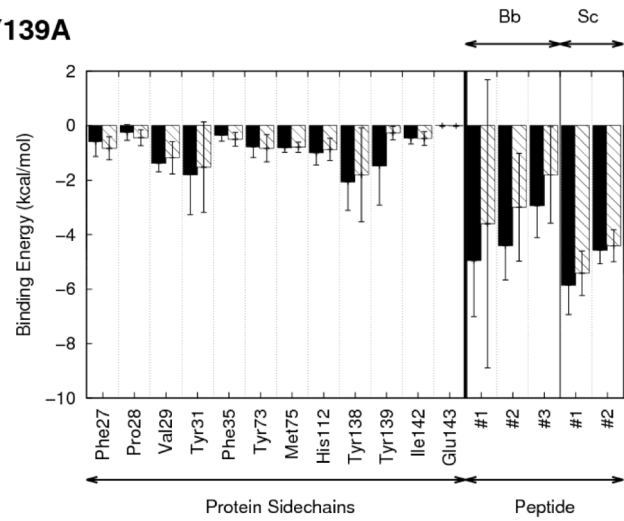

I142A

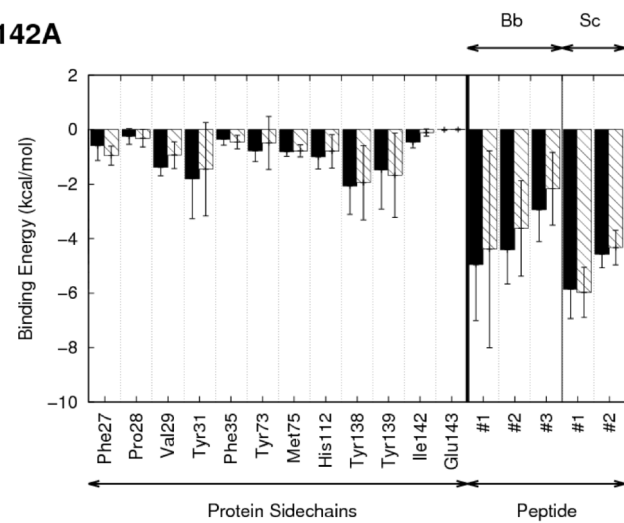

E143A

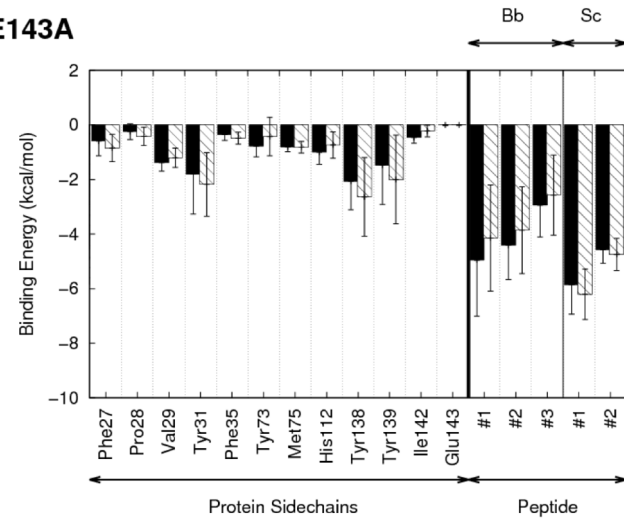

Supplement: Figure S5 — Free energy decompositions (expressed in kcal/mol) of alanine point-mutants of hNaa50p/MLG. Black bars are used for the wild-type hNaa50p/MLG and white dashed bars for the mutant (F27A, P28A, V29A, Y31A, F35A, Y73A, H112A, Y139A, I142A). (PDF) [file pone.0052642.s005.pdf]

## P28A

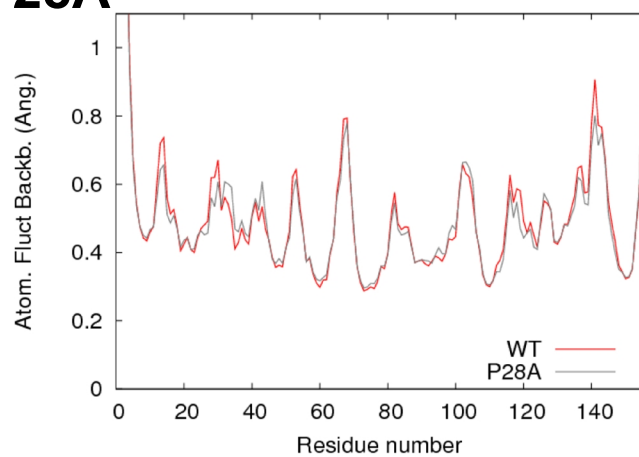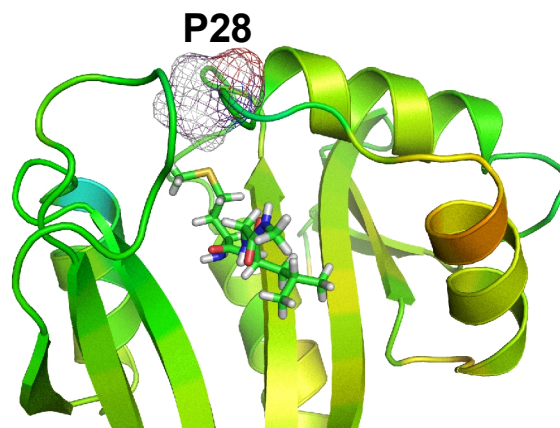

## V29A

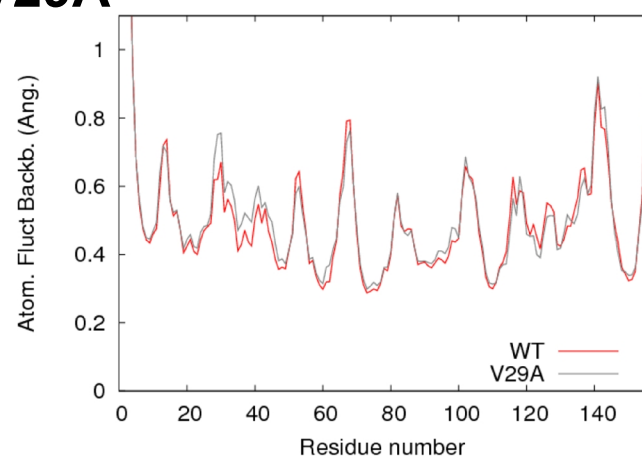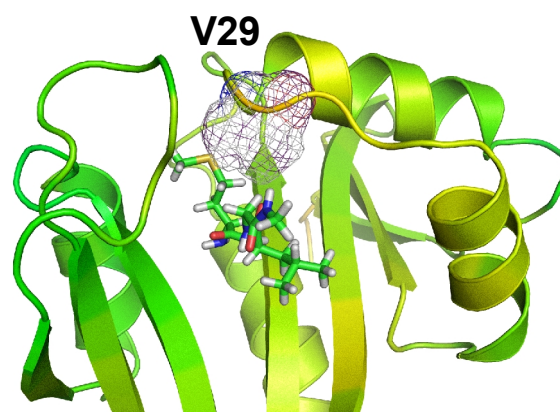

## Y31A

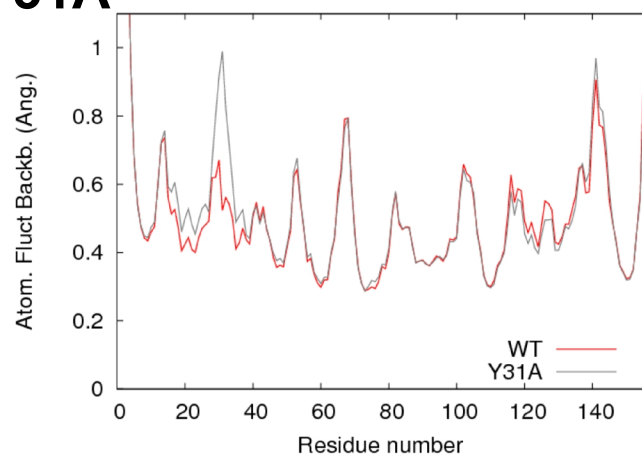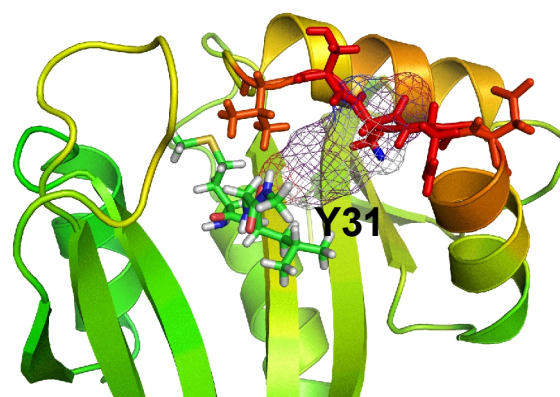

+40% 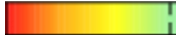 -40%

## F35A

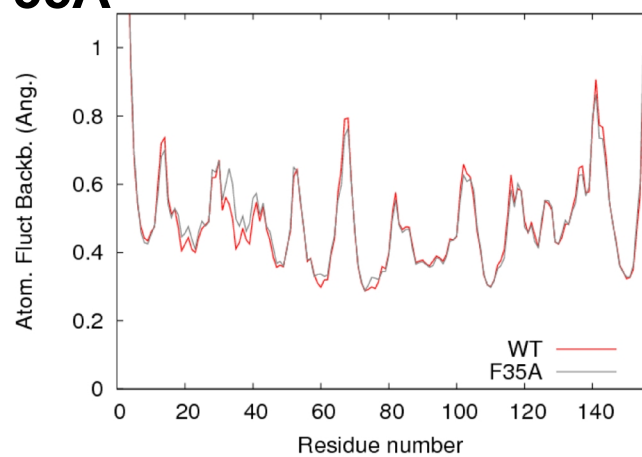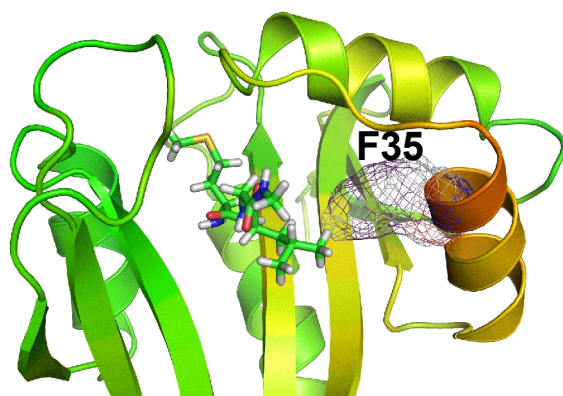

## Y73A

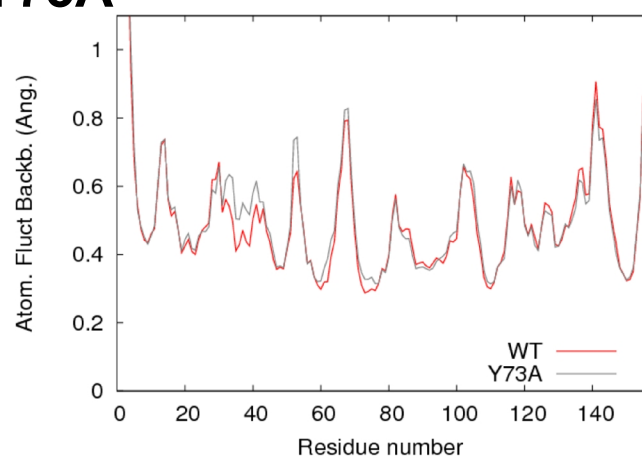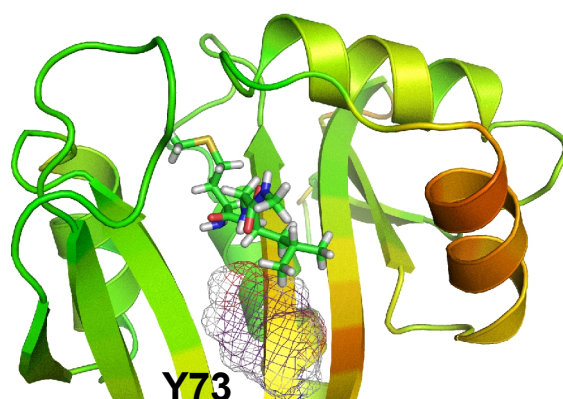

## H112A

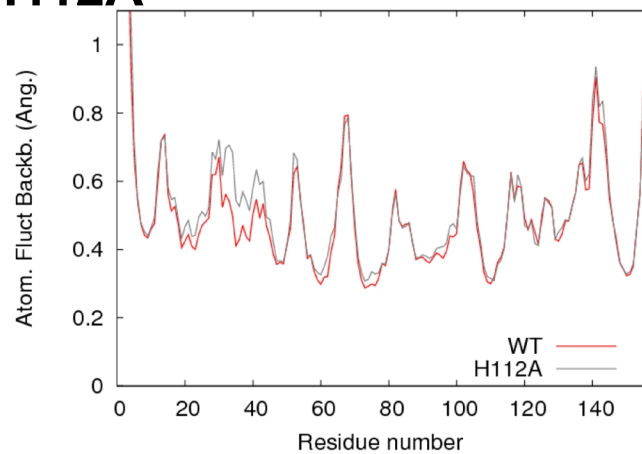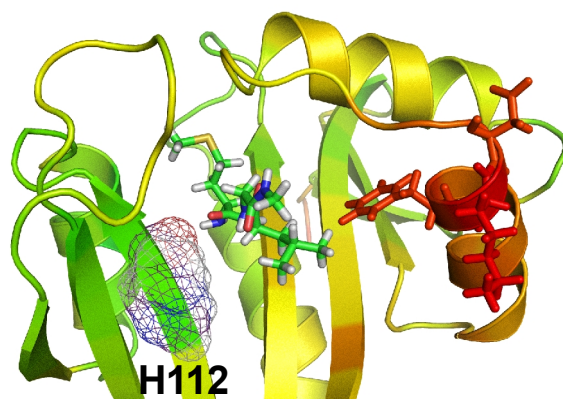

+40% 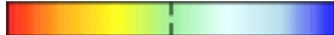 -40%

## Y139A

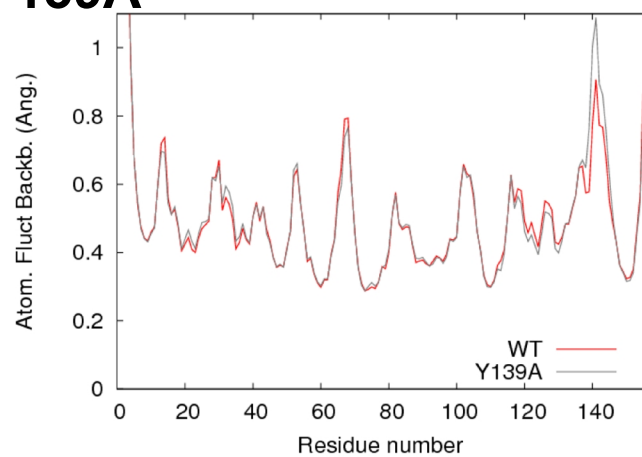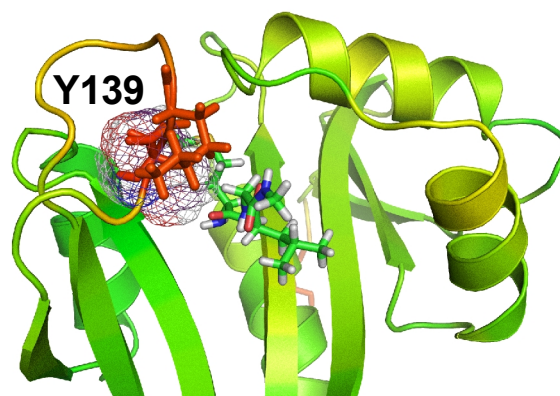

## I142A

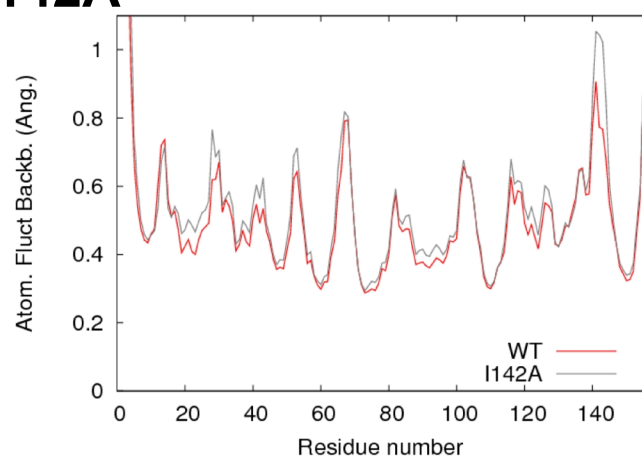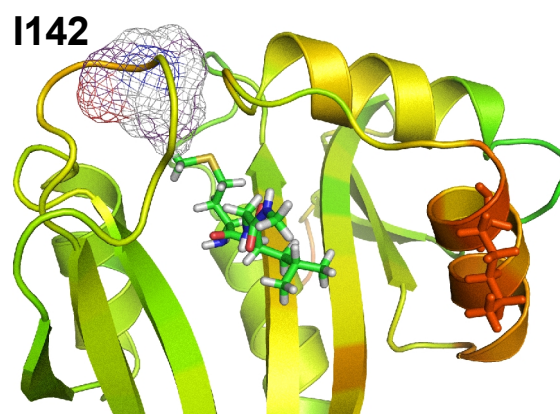

+40% 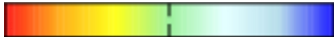 -40%

Supplement: Figure S6 — Change in backbone atomic fluctuations of hNaa50p/MLG resulting from alanine point-mutations. Plots (left) compare the atomic fluctuations of the mutant (P28A, V29A, Y31A, F35A, Y73A, H112A, Y139A, I142A) and wild-type, while the relative difference is shown on the structure (right). Amino acids of the protein are shown in sticks if the difference of fluctuations exceeds 30%. The scale goes from −40% (blue : decreased flexibility) to +40% (red : increased flexibility). (PDF) [file pone.0052642.s006.pdf]

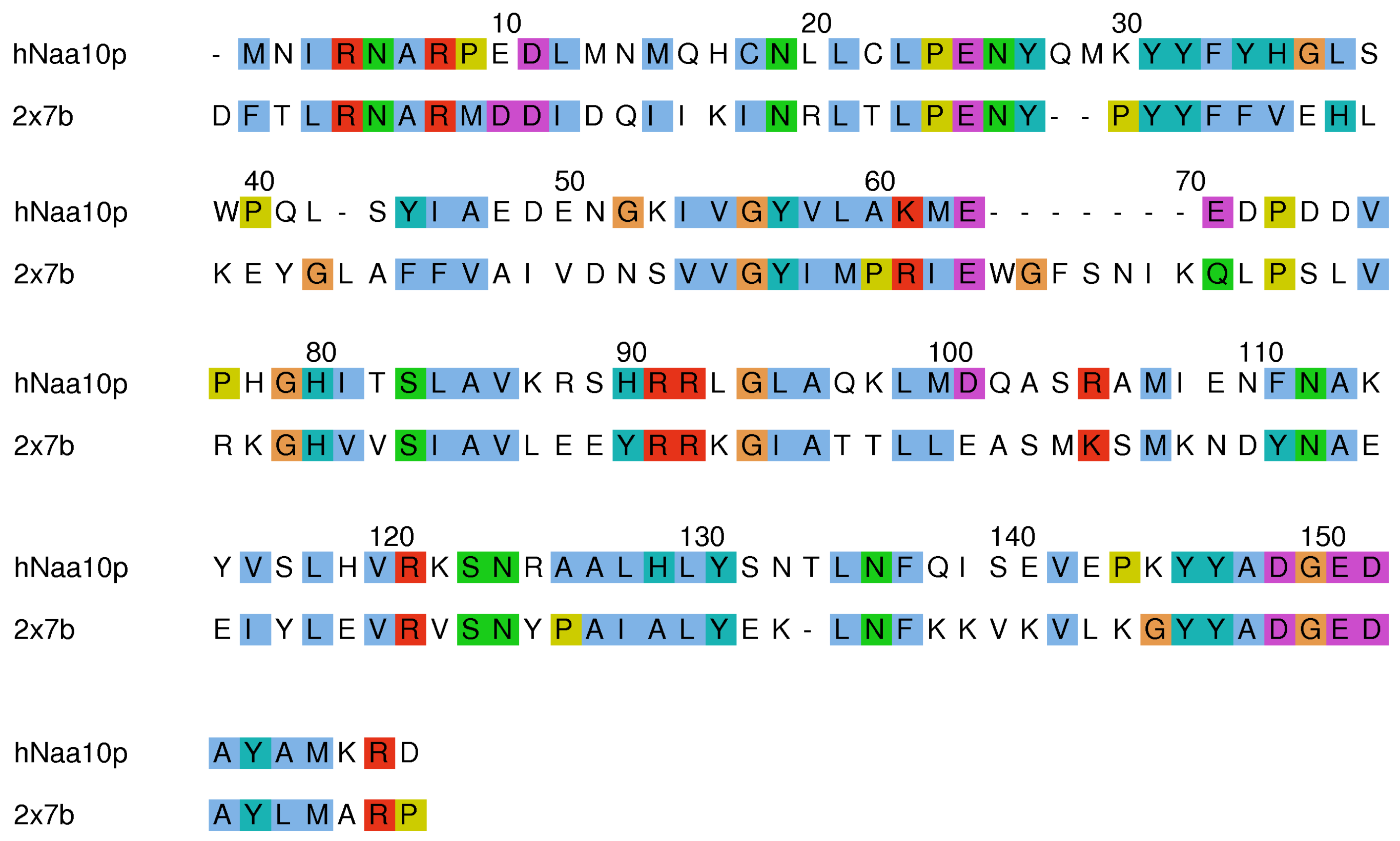

Supplement: Figure S7 — Sequence alignment between hNaa10p and the template from Sulfolobus Solfataricus (PDBid 2X7B ) (TIF) [file pone.0052642.s007.tif]

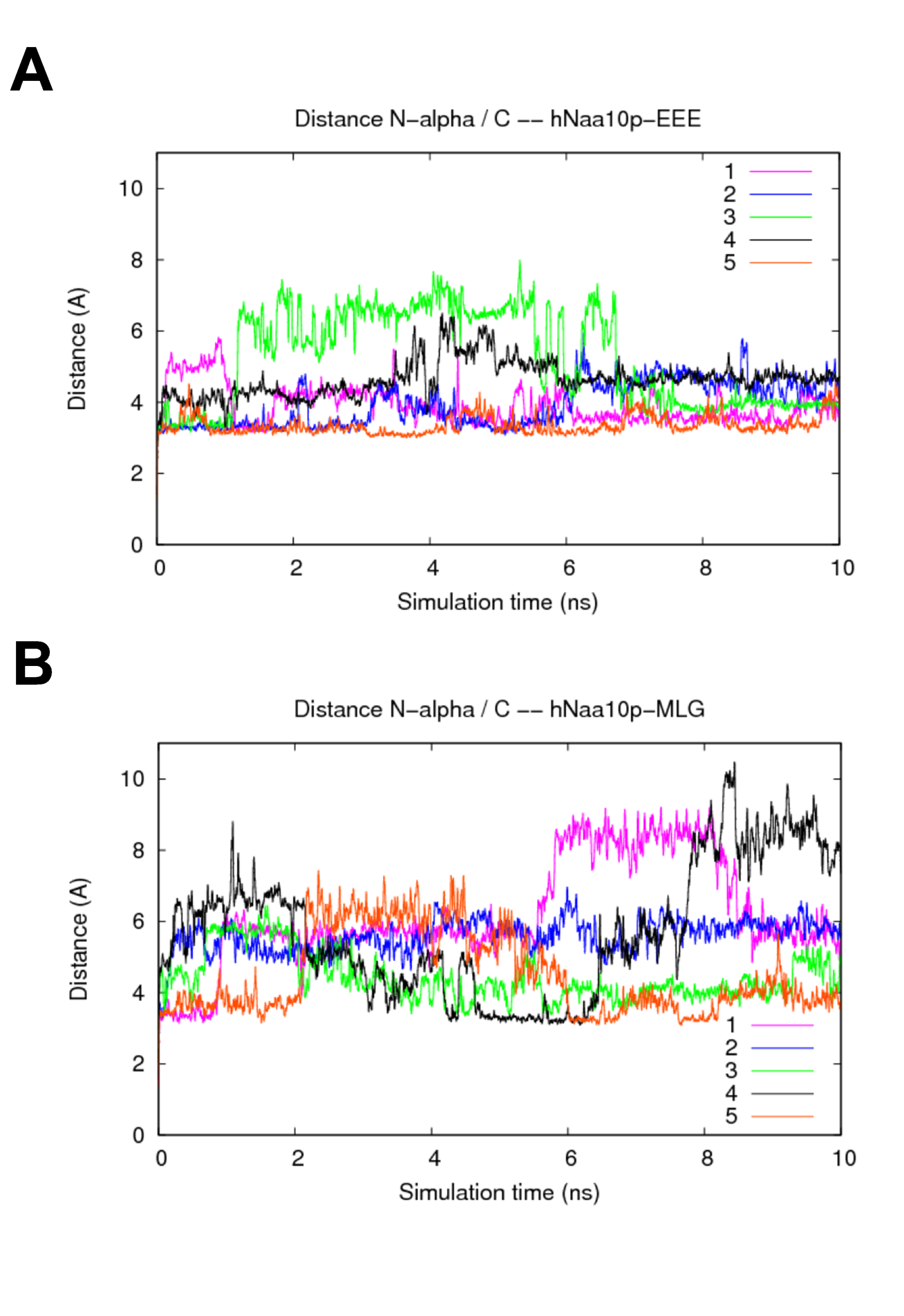

Supplement: Figure S8 — Distance (Å) between the N-terminal nitrogen of Met1 and the carbon atom of Ac-CoA carbonyl in hNaa10p complexed with EEE (top) and MLG (bottom). In each case we represent the evolution of the distance for the five simulations of the corresponding complex. (TIF) [file pone.0052642.s008.tif]
