# Supplementary material for: Specificity and Versatility of Substrate Binding Sites in Four Catalytic Domains of Human N-Terminal Acetyltransferases
Source: PLoS One. 2012 Dec 28;7(12):e52642. doi: 10.1371/journal.pone.0052642 (PMC3532069; doi:10.1371/journal.pone.0052642)
Supplement: Figure S9 — CHARMM force field parameters for AcCoA. Parameters provided in the CHARMM topology format. (PDF) [file pone.0052642.s009.pdf]

```
   * Topology file for Acetyl CoEnzyme A
*

   read rtf card append
* topology for Acetyl CoEnzyme A
*
31 1
   DEFA FIRS NONE LAST NONE
AUTO ANGLES DIHE

   RESI ACO -3.0 !  Acetyl CoEnzyme A
GROUP
ATOM C4B CN7 0.16
ATOM H4B HN7 0.09
ATOM O4B ON6B -0.50
ATOM C1B CN7B 0.16
ATOM H1B HN7 0.09
GROUP
ATOM C5A CN5 0.28
ATOM N7A NN4 -0.71
ATOM C8A CN4 0.34
ATOM H8A HN3 0.12
ATOM N9A NN2 -0.05
ATOM N1A NN3A -0.74
ATOM C2A CN4 0.50
ATOM H2A HN3 0.13
ATOM N3A NN3A -0.75
ATOM C4A CN5 0.43
ATOM C6A CN2 0.46
ATOM N6A NN1 -0.77
ATOM H61 HN1 0.38
ATOM H62 HN1 0.38
GROUP
ATOM C2B CN7B 0.14
ATOM H2'' HN7 0.09
ATOM O2B ON5 -0.66
ATOM H2B HN5 0.43
GROUP
ATOM C3B CN7 0.01
ATOM H3B HN7 0.09
ATOM P3B P 1.50
ATOM O7A ON3 -0.82
ATOM O8A ON3 -0.82
ATOM O3B ON2 -0.62
ATOM O9A ON4 -0.68
ATOM H9A HN4 0.34
```

```
GROUP
ATOM C5B CN8B -0.08
ATOM H5' HN8 0.09
ATOM H5'' HN8 0.09
ATOM O5B ON2 -0.62
ATOM P1A P 1.50
ATOM O1A ON3 -0.82
ATOM O2A ON3 -0.82
ATOM O3A ON2 -0.68
ATOM P2A P 1.50
ATOM O4A ON3 -0.82
ATOM O5A ON3 -0.82
ATOM O6A ON2 -0.62
ATOM CCP CN8B -0.08
ATOM HCP1 HN8 0.09
ATOM HCP2 HN8 0.09
GROUP
ATOM CBP CT2 0.00
ATOM CDP CT3 -0.27
ATOM HDP1 HA 0.09
ATOM HDP2 HA 0.09
ATOM HDP3 HA 0.09
ATOM CEP CT3 -0.27
ATOM HEP1 HA 0.09
ATOM HEP2 HA 0.09
ATOM HEP3 HA 0.09
GROUP
ATOM CAP CT2 0.14
ATOM HAPC HA 0.09
ATOM OAP OH1 -0.66
ATOM HAPO H 0.43
GROUP
ATOM C9P C 0.51
ATOM O9P O -0.51
GROUP
ATOM N8P NH1 -0.47
ATOM H8P H 0.31
ATOM C7P CT2 -0.02
ATOM H7P1 HB 0.09
ATOM H7P2 HB 0.09
GROUP
ATOM C6P CT2 -0.18
ATOM H6P1 HA 0.09
ATOM H6P2 HA 0.09
GROUP
ATOM C5P C 0.51
```

```
ATOM O5P O -0.51
GROUP
ATOM N4P NH1 -0.47
ATOM H4P H 0.31
ATOM C3P CT2 -0.02
ATOM H3P1 HB 0.09
ATOM H3P2 HB 0.09
GROUP
ATOM C2P CT2 -0.20
ATOM H2P1 HA 0.09
ATOM H2P2 HA 0.09
ATOM S1P S 0.06
ATOM C C 0.45
ATOM O O -0.49
GROUP
ATOM CH3 CT3 -0.27
ATOM HH31 HA 0.09
ATOM HH32 HA 0.09
ATOM HH33 HA 0.09

   BOND N6A H61 N6A H62 N6A C6A C6A C5A
BOND N1A C2A C5A N7A C8A H8A
BOND C8A N9A N9A C4A C4A N3A C2A H2A
BOND N9A C1B C1B H1B C1B O4B C1B C2B C2B H2''
BOND C2B O2B O2B H2B C2B C3B C3B C4B C3B O3B
BOND C3B H3B C4B O4B C4B H4B O3B P3B P3B O8A
BOND P3B O9A P3B O7A O9A H9A C4B C5B C5B H5'
BOND C5B H5'' C5B O5B O5B P1A P1A O1A P1A O2A
BOND P1A O3A O3A P2A P2A O4A P2A O5A P2A O6A
BOND O6A CCP CCP HCP1 CCP HCP2 CCP CBP CBP CDP
BOND CBP CEP CBP CAP CDP HDP1 CDP HDP2 CDP HDP3
BOND CEP HEP1 CEP HEP2 CEP HEP3 CAP HAPC CAP OAP
BOND OAP HAPO CAP C9P C9P N8P N8P H8P
BOND N8P C7P C7P H7P1 C7P H7P2 C7P C6P C6P C5P
BOND C6P H6P1 C6P H6P2 C5P N4P N4P H4P
BOND N4P C3P C3P H3P1 C3P H3P2 C3P C2P C2P S1P
BOND C2P H2P1 C2P H2P2 S1P C C CH3
BOND CH3 HH31 CH3 HH32 CH3 HH33
DOUBLE N1A C6A N3A C2A C4A C5A N7A C8A
DOUBLE C9P O9P C5P O5P C O
DONO H61 N6A
DONO H62 N6A
DONO H2B O2B
DONO H9A O9A
DONO H4P N4P
DONO H8P N8P
```

```
DONO HAPO OAP
ACCE N3A
ACCE N7A
ACCE N1A
ACCE O2B
ACCE O3B
ACCE O4B
ACCE O5B
ACCE O1A P1A
ACCE O2A P1A
ACCE O5A P2A
ACCE O4A P2A
ACCE O3A
ACCE O6A
ACCE O C
ACCE O5P C5P
ACCE O9P C9P
IMPR N6A C6A H61 H62
IMPR C6A N1A C5A N6A
IMPR N8P C9P C7P H8P
IMPR N4P C5P C3P H4P
IMPR C9P CAP N8P O9P
IMPR C5P C6P N4P O5P
IMPR C CH3 S1P O
PATC FIRS NONE LAST NONE
```
